# The Gut Microbiota in Camellia Weevils Are Influenced by Plant Secondary Metabolites and Contribute to Saponin Degradation

**DOI:** 10.1128/mSystems.00692-19

**Published:** 2020-03-17

**Authors:** Shouke Zhang, Jinping Shu, Huaijun Xue, Wei Zhang, Yabo Zhang, Yaning Liu, Linxin Fang, Yangdong Wang, Haojie Wang

**Affiliations:** a State Key Laboratory of Tree Genetics and Breeding, Chinese Academy of Forestry, Beijing, People’s Republic of China; b Research Institute of Subtropical Forestry, Chinese Academy of Forestry, Hangzhou, Zhejiang, People’s Republic of China; c Key Laboratory of Zoological Systematics and Evolution, Institute of Zoology, Chinese Academy of Sciences, Beijing, People’s Republic of China; USDA-Agricultural Research Service, Boyce Thompson Institute, Cornell University

**Keywords:** *Camellia* weevil, degradation, diversity, gut microbiome, phytophagous insect, plant secondary metabolites, tea saponin

## Abstract

The camellia weevil (CW [Curculio chinensis]) is a notorious host-specific predator of the seeds of *Camellia* species in China, causing seed losses of up to 60%. The weevil is capable of overcoming host tree chemical defenses, while the mechanisms of how these beetles contend with the toxic compounds are still unknown. Here, we examined the interaction between the gut microbes of CW and camellia seed chemistry and found that beetle-associated bacterial symbionts mediate tea saponin degradation. We demonstrate that the gut microbial community profile of CW was significantly plant associated, and the gut bacterial community associated with CW feeding on Camellia oleifera seeds is enriched with genes involved in tea saponin degradation compared with those feeding on Camellia sinensis and Camellia reticulata seeds. Twenty-seven bacteria from the genera Enterobacter, Serratia, Acinetobacter, and Micrococcus subsisted on tea saponin as a sole source of carbon and nitrogen, and Acinetobacter species are identified as being involved in the degradation of tea saponin. Our results provide the first metagenome of gut bacterial communities associated with a specialist insect pest of *Camellia* trees, and the results are consistent with a potential microbial contribution to the detoxification of tree-defensive chemicals.

**IMPORTANCE** The gut microbiome may play an important role in insect-plant interactions mediated by plant secondary metabolites, but the microbial communities and functions of toxic plant feeders are still poorly characterized. In the present study, we provide the first metagenome of gut bacterial communities associated with a specialist weevil feeding on saponin-rich and saponin-low camellia seeds, and the results reveal the correlation between bacterial diversity and plant allelochemicals. We also used cultured microbes to establish their saponin-degradative capacity outside the insect. Our results provide new experimental context to better understand how gut microbial communities are influenced by plant secondary metabolites and how the resistance mechanisms involving microbes have evolved to deal with the chemical defenses of plants.

## Abstract (precis)

The gut microbiome may play an important role in insect-plant interactions mediated by plant secondary metabolites, but the microbial communities and functions of toxic plant feeders are still poorly characterized. In the present study, we provide the first metagenome of gut bacterial communities associated with a specialist weevil feeding on saponin-rich and saponin-low camellia seeds, and the results reveal the correlation between bacterial diversity and plant allelochemicals. We also used cultured microbes to establish their saponin-degradative capacity outside the insect. Our results provide new experimental context to better understand how gut microbial communities are influenced by plant secondary metabolites and how the resistance mechanisms involving microbes have evolved to deal with the chemical defenses of plants.

## INTRODUCTION

The coevolutionary interactions between plants and herbivores are commonly mediated by morphological and chemical defensive traits (1–3). Plant structural traits such as toughness, latex, trichomes, surface waxes, and plant architecture can make it difficult for arthropod pests to access or process foliage (1, 4). Some secondary metabolites, e.g., alkaloids, terpenoids, cardenolides, glucosinolates, and oxalates, are toxic, antinutritive, or repellent for herbivores, thus acting as defensive compounds (5–8). Such insect-plant interactions are important for understanding plasticity in the herbivore diet, host preference, as well as impacts on host growth and performance (1, 5, 9), and a clear understanding of the underlying defense mechanisms of plants is crucial for exploiting plant-defensive traits in crop breeding to manage insect pests (3, 10).

It is widely recognized that secondary metabolites are fundamental to the plant defense against arthropod herbivores. Secondary compounds are involved in resistance to most insects and susceptibility to others (1, 2, 11, 12). Saponins are a class of secondary plant metabolites that includes triterpenoids, steroids, and steroidal alkaloids glycosylated with one or more sugar chains. Saponins in plants taste bitter, and they have been hypothesized to exert a repellent/deterrent activity, give rise to digestive problems, provoke molting defects, and exert toxic effects in insects (13–16). Saponins are produced by many plant species. The plants in the genus Camellia represent a particularly rich source of triterpene saponins (ca. 10 to 30% of seed dry weight).

The genus *Camellia* comprises economically and nutritionally important perennial monoculture crops, the most well known being C. sinensis, C. oleifera, and C. japonica. The leaves are used to produce tea, and the seeds are used to manufacture cooking oil for human consumption. The plants are thus commonly referred to as “tea tree” or “tea shrub.” Tea is widely cultivated in more than 34 countries across Asia, Africa, and Latin America. To date, more than 1,000 arthropod species exploit *Camellia* plants as a food resource, but due to high contents of saponins and other bioactive chemicals such as caffeine, theanine, and epigallocatechin gallate (EGCG), few species can utilize the seeds. However, the camellia weevils, Curculio spp. (Coleoptera: Curculionidae), represent striking exceptions to this rule, as they can feed and complete the larval stage solely in the seeds.

The camellia weevil (CW), Curculio chinensis Chevrolat, is a notorious host-specific predator of the seeds of *Camellia* trees in China (17–19). Adult camellia weevils typically emerge from the soil during late April to May and feed on unripe fruits for supplemental nutrition. After successfully mating, female beetles deposit eggs in the *Camellia* seeds. Larvae feed on the seeds before leaving the host plant, causing significant losses both in quality and yields (17, 18). Yearly economic losses inflicted by CW have been estimated at over $1.4 billion in China (17, 20). The cryptic life cycle inside the camellia seeds and soil makes CW control difficult. Previous work has shown that some *Camellia* species, including *C. reticulata*, C. chekiangoleosa, and *C. japonica*, showed possible resistance to CW (17, 21). However, detailed information on the defense mechanisms is scarce.

Empirical evidence has demonstrated that variation in plant defense chemical levels can impact the host preference, growth, and performance of arthropod herbivores (2, 22). Although variation in the saponin concentrations of different *Camellia* species has been observed, the correlation between saponin content in seeds of *Camellia* species and resistance to *C. chinensis* remains unclear. CW larvae feed on a broad variety of camellia plants, suggesting that the camellia weevil is adapted to a wide range of saponin concentrations. At present, the mechanism camellia weevils employ to overcome the plant biochemical defense barriers is unknown.

Over time, herbivores have evolved different mechanisms to circumvent the noxious effects of plant defenses (8). One of the strategies that insects have used to break down dietary deterrents and/or toxins is enlisting the cooperation of microbes. Recent compelling evidence suggests that gut microbiota in herbivorous insects are prominent mediators aiding in the detoxification of plant allelochemicals, thus conferring resistance to plant defenses (5, 9, 23). Some recent examples are terpene detoxification in pine pests (8), caffeine detoxification in the coffee berry borer (6), oleuropein detoxification in the olive fruit fly (24), and isothiocyanate detoxification in the cabbage root fly (23). Camellia weevils have the ability to disturb host tree defenses, and they can tolerate highly toxic levels of triterpene saponins. Unfortunately, to date, even basic information on camellia weevil microbial symbiosis is lacking. Whether the gut microbiota of camellia weevils is involved in overcoming the toxic compounds in camellia seeds is unknown.

Here, we examined the interaction between CW gut microbes and camellia seed chemistry. We hypothesized that variation in tea saponin levels affected the intestinal bacterial community of camellia weevil larvae and that the gut microbiota could mediate tea saponin degradation. We assessed the *in vitro* impacts of five different secondary metabolites in camellia seeds on the gut bacterial community of camellia weevil larvae. In addition, we monitored the development of larvae in the seeds of three *Camellia* species with different saponin content in the seeds. Moreover, we present the first study of the gut microbial community profiling of CW feeding on the camellia seeds with different saponin levels. We also used cultured camellia weevil microbes to establish their tea saponin-degradative capacity outside the insect. Our findings show that the microbial community associated with the gut of the insect is significantly influenced *in vivo* by its diet, and that the microbiota component mediates effective tea saponin degradation in the camellia weevil. Our results may enhance our understanding of the interactions between insect gut microbiota and plant toxins and may lead to the development of new sustainable environment-friendly symbiont-based pest control strategies.

## RESULTS

### Effects of five plant chemicals on the intestinal microbial diversity of CW larvae.

To test the hypothesis that plant defense chemicals are involved in resistance to arthropod herbivores by mediating the intestinal microbial community, we assessed the *in vitro* impact of five secondary metabolites in camellia seeds and their concentrations on the gut bacterial community of camellia weevil larvae. First, we extracted genomic DNA from a total of 205 samples and obtained 6,828,129 bp of 16S rRNA sequences. Thereafter, all high-quality sequences were assigned to 142,093 operational taxonomic units (OTUs) at the level of 97% similarity. Rarefaction curves of all 205 samples for 16S rRNA gene sequencing tended to be saturated (see Fig. S1 in the supplemental material), indicating that the sequencing depths for these specimens were appropriate. There was no abundance of unclassified bacteria for all experimental treatments.

10.1128/mSystems.00692-19.1 FIG S1 Rarefaction curve of 16S rRNA in the gut microbiome of *Curculio chinensis* Chevrolat in five treatments. Download FIG S1, PDF file, 0.5 MB. Copyright © 2020 Zhang et al. 2020 Zhang et al. This content is distributed under the terms of the Creative Commons Attribution 4.0 International license.

**FIG 1:**
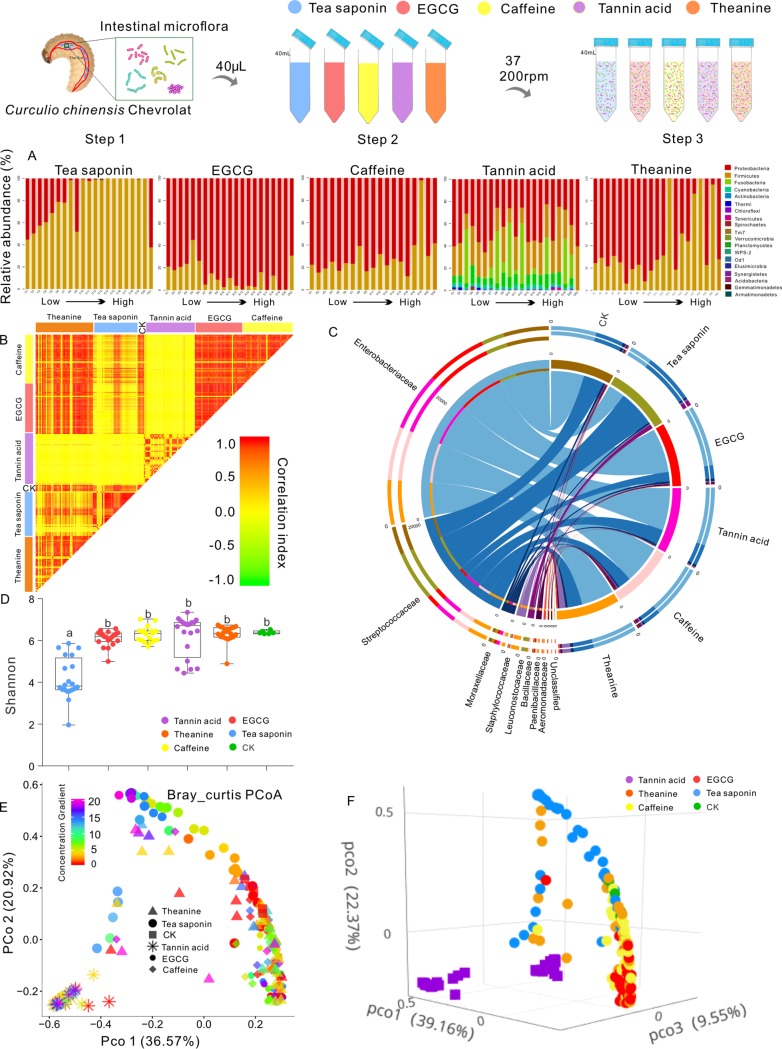
Gut microbial communities of *Curculio chinensis* larvae are influenced by plant secondary metabolite and chemical concentrations. Steps 1 to 3 show the specific experimental process. (A) Histograms of phylum abundances of the *in vitro*-cultured gut microbiota in each treatment and control. Shown are changes in the relative abundances of bacterial phyla by low-concentration samples to high-concentration samples in the five treatment groups. The figure shows 20 replicate samples. (B) Pairwise correlation analysis for five chemicals of five intertreatment and intratreatment samples. (C) Microbial compositions of the five samples shown at the family level. (D) Shannon index analysis based on unweighted UniFrac distance for the samples receiving one of the five treatments. The horizontal bars within boxes represent medians. The tops and bottoms of the boxes represent the 75th and 25th percentiles, respectively. The upper and lower whiskers extend to data no more than 1.5× the interquartile range from the upper edge and lower edge of the box, respectively. Bars with different lowercase letters differ significantly at *P* = 0.05. (E) Principal-coordinate analysis plot of 16S rRNA gene weighted Bray-Curtis distances for the five treated and control groups (*P* < 0.001, permutational multivariate analysis of variance [PERMANOVA] by Adonis). (F) Three-dimensional (3D) display for principal-coordinate analysis plot of 16S rRNA gene weighted Bray-Curtis distances (*P* < 0.001, PERMANOVA by Adonis).

Compared to the control, the observed richness of the CW gut bacterial communities as indicated by the number of observed OTUs and dominant bacterial classes varied with five plant chemical treatments (Fig. 1A and S2). The top bacterial families were identified by applying random forest classification of the relative abundances of the gut microbiota in the five-treatment samples. *Bacillus*, *Gammaproteobacteria*, and *Betaproteobacteria* were dominant for the tea saponin and theanine treatments, while *Bacillus* and *Gammaproteobacteria* were predominant in the EGCG and caffeine treatments (Fig. 1A and S2). Moreover, only the tea saponin treatment gut bacterial community showed a significant gradient change within different concentration treatments, and *Deinococcus*, *Actinobacteria*, *Betaproteobacteria*, Ellin6529, chloroplasts, and *Clostridia* showed obvious enrichment (Fig. 1A and S5).

10.1128/mSystems.00692-19.2 FIG S2 The top bacterial families were identified by applying random forest classification of the relative abundances of the gut microbiota in the samples receiving one of the five treatments. Biomarker taxa are ranked in descending order of importance to the accuracy of the model. Download FIG S2, PDF file, 0.1 MB. Copyright © 2020 Zhang et al. 2020 Zhang et al. This content is distributed under the terms of the Creative Commons Attribution 4.0 International license.

Pairwise correlation analysis revealed that the gut microbiota varied dramatically in 24 h for the five chemical treatments with the gradient change in concentrations. The change was significantly different between treatments with tannic acid and tea saponin (Fig. 1B and S3). In addition, we found that the lowest correlation was between high concentration and low concentration for the tea saponin treatment. For other treatments, no significant differences were observed between treatments with different concentrations (Fig. S2).

To evaluate the variation in gut microbiomes with the five treatments, alpha diversity was estimated by four indices, i.e., abundance-based coverage estimator [ACE], Shannon, Simpson, and Chao1 indices. Alpha diversity was significantly different (Welch’s *t* test, Shannon’s index, *P* = 0.0001) between the tea saponin and other treatments (Fig. 1D). The Shannon diversity indices were lowest for the tea saponin treatment (Fig. 1D). However, there were no significant differences in most alpha-diversity indices (Chao1, Shannon, and Simpson) within the theanine, caffeine, and EGCG treatments.

To investigate the impacts of defense chemicals on the gut bacterial community structure of CW larvae, we conducted principal-coordinate analysis (PCoA) using Bray-Curtis dissimilarity distances. Compared with the blank control treatments, the bacterial communities within EGCG, theanine, tannic acid, and caffeine treatment samples were clustered together, and tea saponin samples were shifted far across the chemicals and chemical concentration treated in the first coordinate axis, indicating that chemicals and chemical concentration are the main factors influencing the gut microbiota community (Fig. 1E and F). To better understand the influence and correlation of the five chemicals on intestinal microbial diversity, we constructed a Mantel test matrix between the unweighted UniFrac distance and the concentration differences of the five treatments. Three indices (Spearman, Kendall, and Pearson) showed that the tea saponin treatment had a significant impact on the intestinal microbial diversity of CW larvae (Spearman, Kendall, and Pearson correlation coefficients were calculated, respectively, as 0.0349, *P* < 0.001; 0.2785, *P* < 0.001; and 0.2747, *P* < 0.002) (Table S3).

### Impact of fruit development on larval development.

**FIG 2:**
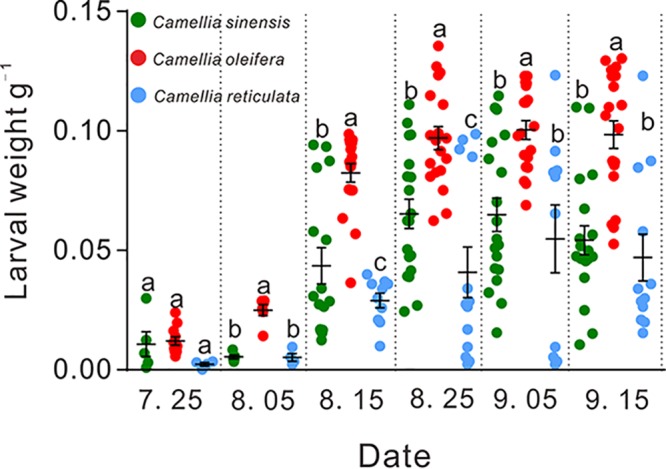
Mean body weights of *Curculio chinensis* larvae developing in three different *Camellia* host seeds. Different colors represent different host trees (green, C. sinensis; red, *C. oleifera*; blue, *C. reticulata*). Each symbol represents a larva from one *Camellia* host. Different lowercase letters indicate significant differences between groups (*P* < 0.001).

To test whether tea saponin content levels could affect larval development *in vivo*, we monitored the development of larvae in the seeds of three species of *Camellia*. Measuring the change in larval weight according to age revealed a significant difference between the three *Camellia* hosts (*C. oleifera*, C. sinensis, and *C. reticulata*) (Fig. 2). As newly hatched larvae, the larval body weight was maintained between 0.0001 and 0.025 g, and no significant difference was found between the three species of host plants (*P* > 0.05, Fig. 2). Before completing development and exiting the fruit as prepupal larvae, the weight of CW larvae was significantly affected by the species of *Camellia*, and the larvae in the *C. oleifera* group developed significantly faster than did those in the other two host groups (*P* < 0.01, Fig. 2). From 25 July to 15 September 2018, on average, larval weight in the *C. oleifera* group increased 7.79 times over the weight of newly hatched larvae, and these larvae were 149% and 238% heavier than those in the C. sinensis group and the *C. reticulata* group, respectively (*C. oleifera*, 0.097 ± 0.048 g; C. sinensis, 0.0653 ± 0.006 g; *C. reticulata*, 0.0408 ± 0.010 g). Remarkably, after 15 September, all of the larvae exited from the seeds of *C. oleifera* for pupating, while few of the larvae fed on the seeds of C. sinensis and *C. reticulata* developed into prepupal larvae and exited from the seeds.

**FIG 3:**
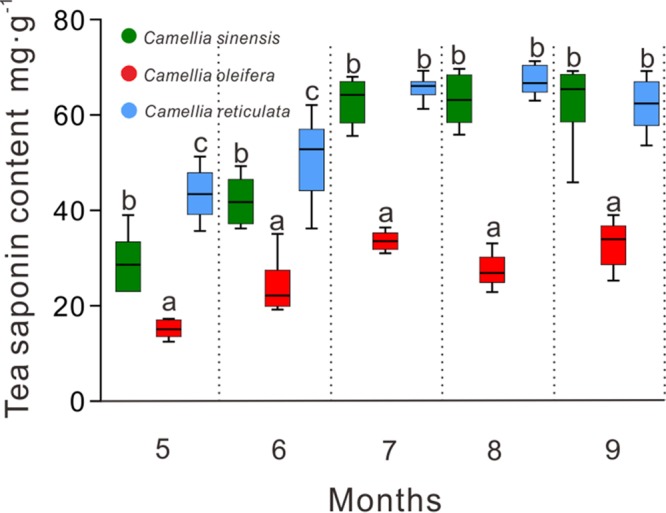
Accumulation of tea saponin in the seeds during the development of three host trees. Different colors represent the larval groups extracted from three hosts (green, C. sinensis; red, *C. oleifera*; blue, *C. reticulata*). Different lowercase letters indicate significant differences between groups (*P* < 0.001).

To test the hypothesis that the variation in CW performance in seeds of different hosts as indicated by difference in body weight was caused by variation in tea saponin concentrations, we measured the host accumulation pattern of tea saponin in the seeds of three camellia trees from May to September. The tea saponin concentrations differed significantly (*P* < 0.001) with the camellia fruit maturation process. The tea saponin content in *C. oleifera* seeds was significantly lower than that in the other two host plants, *C. reticulata* and C. sinensis (*P* < 0.01) (Fig. 3). Nevertheless, the seed saponin contents of the two trees were similar (*P* > 0.05) (Fig. 3). Correlation analysis indicated that body weight of CW larvae was significantly negative correlated with the seed saponin concentration (Spearman, Kendall, and Pearson correlation coefficients were calculated, respectively, as 0.2517, *P* < 0.05; 0.1872, *P* < 0.01; and 0.2913, *P* < 0.01).

### Gut microbial composition of *Curculio chinensis* feeding on different host plants.

**FIG 4:**
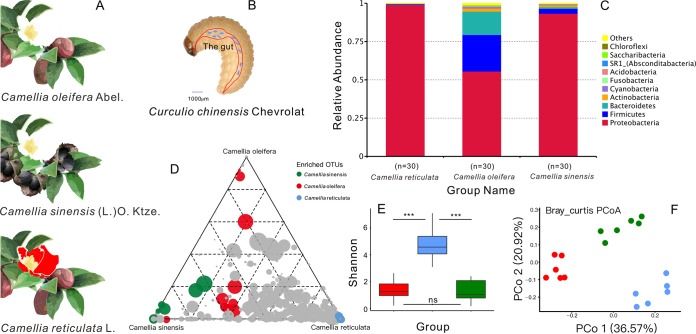
Basic information of the three host plants, the larvae of *Curculio chinensis* Chevrolat, and the gut microbial community of CW larvae feeding on the three host plants. (A) Three host plants of *Camellia*. (B) Physical characteristics of CW larvae. (C) Phylum-level distribution of gut microbiota of CW larvae feeding on the three *Camellia* species. (D) Ternary plot depicting all OTUs (>5‰) found in CW larvae from the three host plants (*n* = 18). Each point corresponds to an OTU. The position of each point represents the RA of the OTU with respect to each compartment, and the size of each point represents the RA (weighted average) across all three compartments. Colored points represent OTUs enriched in one compartment compared with the others (green for C. sinensis group, blue for the *C. reticulata* group, and red for the *C. oleifera* group), whereas gray points represent OTUs that are not significantly enriched in a specific compartment. (E) Shannon index analysis based on unweighted UniFrac distance for the three host plant samples. The horizontal bars within boxes represent medians. The tops and bottoms of the boxes represent the 75th and 25th percentiles, respectively. The upper and lower whiskers extend to data no more than 1.5× the interquartile range from the upper edge and lower edge of the box, respectively. ***, *P* < 0.001; ns, nonsignificant. (F) Principal-coordinate analysis plot of 16S rRNA gene weighted Bray-Curtis distances for the three host plant samples (*P* < 0.001, permutational multivariate analysis of variance [PERMANOVA] by Adonis).

To assess the effect of host plants on gut microbial composition of CW larvae, we sequenced the 16S rRNA genes of CW larvae feeding on different *Camellia* seeds. The genes could be assigned to 35 bacterial phyla (Fig. 4C and S6A to C). There was a relatively low abundance of unclassified bacteria (0.0605%). The top three dominant phyla, accounting for 97.30% of the relative abundance (RA) in all communities, were the *Proteobacteria* (82.67%), *Firmicutes* (9.127%), and *Bacteroidetes* (5.51%). There were high variations in the RA among individuals feeding on different hosts. At the phylum level, samples were dominated by the *Proteobacteria* (*C. oleifera* group, 99.08%; *C. reticulata* group, 55.58%; C. sinensis group, 93.34%), and there was no significant difference (*P* > 0.05) between the *C. oleifera* group and the C. sinensis group (Fig. 4C). The gut microbiomes of both the *C. oleifera* group and the C. sinensis group showed obvious enrichment of the *Proteobacteria*. The gut microbiota of the *C. reticulata* group was enriched in the *Firmicutes* and *Proteobacteria* (Fig. S6D and E). Larvae fed on different *Camellia* seeds shared a common core microbiome but also harbored unique microbes (Fig. 4D).

To evaluate the variation in the gut microbiota of CW larvae fed on the three host plant species, alpha diversity was estimated by four indices (ACE, Shannon, Simpson, and Chao1). Alpha diversity was significantly different between *C. oleifera* and *C. reticulata* (Welch’s *t* test, Shannon, *P* = 0.0001; Chao1, *P* = 0.0007) (Fig. 4E and Table S4). PCoA revealed that the bacterial communities associated with larvae fed on the same plant species were found in close proximity to one another (Fig. 4F). Bacterial communities of *C. oleifera-*fed larvae and those of C. sinensis-fed larvae were closely clustered together; however, those larvae fed on *C. reticulata* were separated (Fig. 4F). Likewise, the results were confirmed by dissimilarity tests of gut community structure. The structures of the gut microbiomes of CW larvae feeding on *C. oleifera*, *C. reticulata,* and C. sinensis differed significantly (Table S1). In comparisons of the bacterial communities, the differences between the *C. oleifera* group and the *C. reticulata* group were greater (for *C. oleifera* group versus *C. reticulata* group, analysis of similarity [ANOSIM], *R* = 0.9 > 0.75, *P* = 0.003; PERMANOVA, *R*^2^ = 0.235, *P* = 0.001; and for the C. sinensis group versus *C. reticulata*, ANOSIM, *R* = 0.5907 > 0.5, *P* = 0.001; PERMANOVA, *R*^2^ = 0.338, *P* = 0.003) (Fig. 4E and Table S1). The PCoA plot also revealed significant interindividual variation among the *C. oleifera*, *C. reticulata,* and C. sinensis groups, indicating that the gut microbiome varied widely among the three species of host plants with different saponin contents in seeds (Fig. 4E).

10.1128/mSystems.00692-19.7 TABLE S1 Dissimilarity tests of *Curculio chinensis* Chevrolat microbial communities using ANOSIM and PERMANOVA based on Bray-Curtis distance. Download Table S1, DOCX file, 0.01 MB. Copyright © 2020 Zhang et al. 2020 Zhang et al. This content is distributed under the terms of the Creative Commons Attribution 4.0 International license.

10.1128/mSystems.00692-19.8 TABLE S2 Basic information of phylogenetic molecular ecological networks (pMENs). Download Table S2, DOCX file, 0.01 MB. Copyright © 2020 Zhang et al. 2020 Zhang et al. This content is distributed under the terms of the Creative Commons Attribution 4.0 International license.

10.1128/mSystems.00692-19.9 TABLE S3 Relationships between pairwise differences (Bray-Curtis distance) in gut bacterial community composition and concentration differences. Download Table S3, DOCX file, 0.1 MB. Copyright © 2020 Zhang et al. 2020 Zhang et al. This content is distributed under the terms of the Creative Commons Attribution 4.0 International license.

10.1128/mSystems.00692-19.10 TABLE S4 Analysis of differences between the alpha-diversity index groups. Download Table S4, DOCX file, 0.1 MB. Copyright © 2020 Zhang et al. 2020 Zhang et al. This content is distributed under the terms of the Creative Commons Attribution 4.0 International license.

In addition to the alpha diversity, differentially represented OTUs were analyzed via a linear discriminant analysis effect size (LEfSe) algorithm, and a statistical measure was used in metagenomic biomarker discovery. According to this analysis, 32 OTUs were identified to be responsible for discriminating between the different gut microbiomes of the CW larvae feeding on the three host plant species (Fig. S6D and E). Notably, this analysis revealed that the dominant intestinal microbial species were plant associated in CW larvae.

### Tea-saponin-degrading microbiomes in the gut of CW larvae.

**FIG 5:**
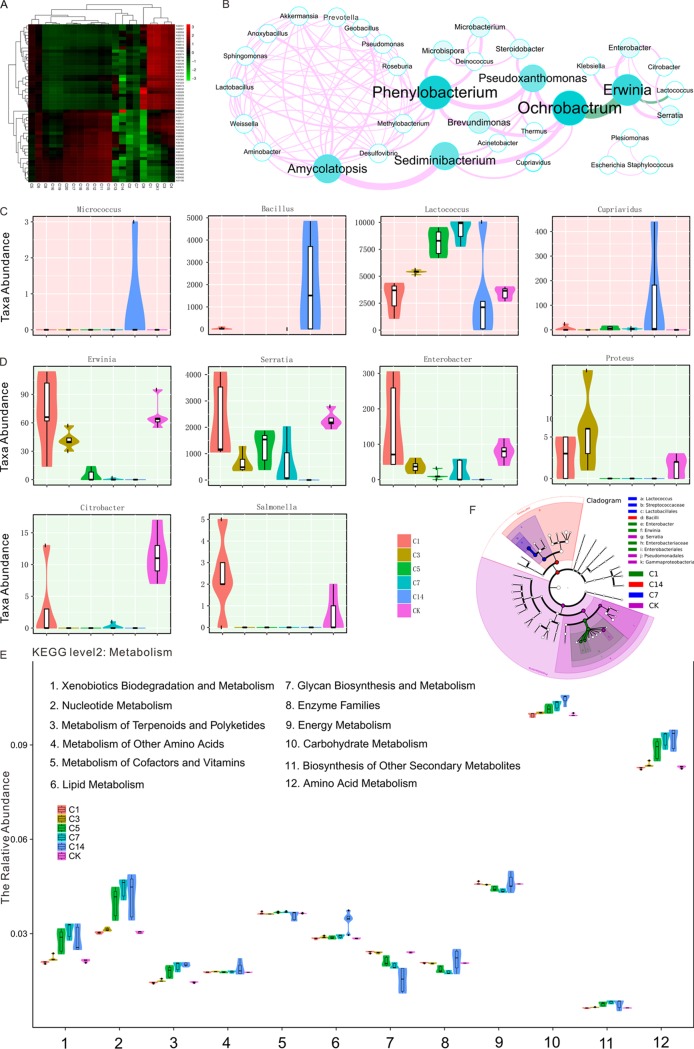
Analysis of the key flora of tea saponin treatment groups. (A) Cluster analysis and heat map of the top 50 functional groups. (B) Network diagram of bacterial groups associated with tea saponin degradation. The size of each node is proportional to the number of connections. The thickness of each connection between two nodes is proportional to the absolute value of Spearman correlation coefficients ≥0.6. (C and D) Abundance distribution maps of taxa with the most significant differences between groups. (E) Second rank distribution map of KEGG predictions. (F) LEfSe showing comparison of tea saponin treatment samples at all levels. The module plots the biomarkers found by LEfSe, ranking them according to four effect sizes and associating them with the class with the highest median. This module produces cladograms representing the LEfSe results on the hierarchy induced by the label names.

To identify members of the CW gut microbiota that were involved in the degradation of tea saponin, cluster analysis was performed, and heat maps of the top 50 functional groups were constructed. We found that 23 OTUs were significantly enriched on the medium-to-high concentrations of tea saponin (Fig. 5A). Network analysis showed that Phenylobacterium, Amycolatopsis, Sediminibacterium, and Ochrobactrum were the predominant groups affiliated with tea saponin degradation in CW, and there was mutual inhibition between Erwinia, *Ochrobactrum*, and Lactococcus spp. (Fig. 5B). We further tested the abundance distribution of each classification unit present in each group through Metastats pawn comparison (*P* < 0.05). The abundances of four groups (Micrococcus, Bacillus, Lactococcus, and Cupriavidus) increased along with the increase in tea saponin concentration, and the abundances of six groups (*Erwinia*, Serratia, Enterobacter, Proteus, Citrobacter, and Salmonella) decreased with the increase in tea saponin concentration (Fig. 5C and D). The LEfSe statistical analysis also showed that unique bacterial groups were detected corresponding to the change in tea saponin concentration (Fig. 5F). To better reveal the relationship between the bacterial community and tea saponin degradation, we conducted KEGG pathway analysis and found that 12 pathways were annotated (Fig. 5E). With the increase in tea saponin concentration, a set of metabolic pathways was indicated, including xenobiotic biodegradation and metabolism, nucleotide metabolism, terpenoid and polyketide metabolism, amino acid metabolism, lipid metabolism, carbohydrate metabolism, biosynthesis of other secondary metabolites, and amino acid metabolism. There were also a multitude of repressed pathways, namely, cofactors and vitamins, glycan biosynthesis, and metabolism. Nevertheless, the enzyme families and energy metabolism were in a complex regulatory relationship (Fig. 5E).

**FIG 6:**
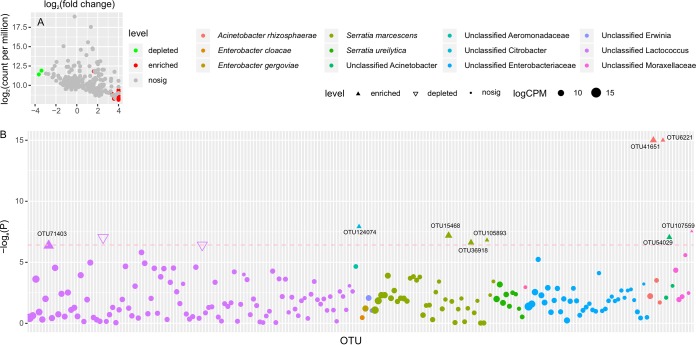
Certain OTUs contribute to tea saponin degradation. (A) Enrichment and depletion of the 209 OTUs included in the tea saponin treatments compared with controls, as determined by differential abundance analysis. Each point represents a single OTU, and the position along the *x* axis represents the abundance fold change compared with the control. Red points represent OTUs enriched and green points represent OTUs depleted, whereas gray points represent OTUs that are not significantly enriched. nosig, nonsignificant; CPM, count per million (reads). (B) Manhattan plots showing enriched OTUs in the tea saponin treatments with respect to the control. CZ means tea saponin treatment groups, and CK represents the control groups. OTUs that are significantly enriched are depicted as filled triangles, and OTUs that are significantly depleted are depicted as open triangles. The dashed line corresponds to the false-discovery rate-corrected *P* value threshold of significance (*P* < 0.05). The color of each dot represents the different taxonomic affiliation of the OTUs (species level), and the size corresponds to their RA in the respective samples.

To determine the core microorganisms contributing to tea saponin degradation, we used a Manhattan diagram to display the differences in OTUs and taxonomy. In the tea saponin treatment groups, 209 OTUs were screened. Nine OTUs were significantly enriched (three for Serratia marcescens; one each for unclassified *Lactococcus*, unclassified *Aeromonadaceae*, Serratia ureilytica, and unclassified Acinetobacter; and two for Acinetobacter rhizosphaerae), and two OTUs (OTU18713 and OTU61711 for unclassified *Lactococcus*) were significantly depleted, as well as several other nonsignificant OTUs (Fig. 6).

### Tea saponin degradation capacity of multistrain and single-strain cultures.

**FIG 7:**
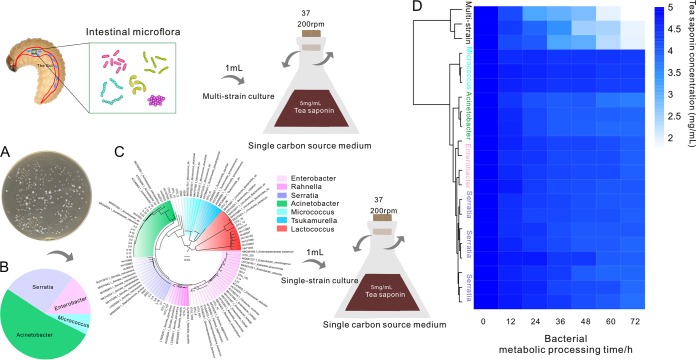
Determination of tea saponin degradation capacity of multistrain and single-strain cultures. (A) Tea saponin gradient plate depicting bacterial colonies isolated from the CW gut. (B) Identification of the bacteria isolated from tea saponin medium by phylogenetic analysis. (C) Phylogenetic tree for the bacteria isolated from tea saponin medium. (D) The capability of different cultures to degrade tea saponin as illustrated using HPLC measurements. Statistical analysis was conducted by one-way ANOVA, followed by a multiple-comparison test using least-significant difference (LSD) at a *P* value of <0.05. Bars marked with different lowercase letters are significantly different at a *P* value of <0.05.

To determine whether the screened gut bacteria were involved in tea saponin degradation in CW, we sequenced the genes of the V3-V4 region extracted from the OTUs and *in vitro*-cultured strains based on the ternary plot (Fig. 2D) and the Manhattan analysis (Fig. 6), and the sequences of the species with the closest relatives were obtained from the NCBI. The annotation of all the OTUs and the single strain could be classified into seven clusters (*Enterobacter*, Rahnella, *Serratia*, Acinetobacter, *Micrococcus*, Tsukamurella, and *Lactococcus*) (Fig. 7C). On solid medium with tea saponin as the single source of carbon and nitrogen, 27 strains could be isolated, of which 14.8% were *Enterobacter* spp., 25.9% were *Serratia* spp., 51.8% were Acinetobacter spp., and the rest were *Micrococcus* spp. (Fig. 7B). The results of tea saponin degradation by cultured microbes showed that the degradation capacity of the multistrain cultures was the strongest (residual tea saponin content, 1.867 ± 0.066 mg/ml in 72 h), which was significantly faster (*P* < 0.001) than the degradation of cultures treated with CK (residual tea saponin content, 4.054 ± 0.012 mg/ml in 72 h) or other single-bacterium-strain cultures (Fig. 7D). The degradation rate of the treatment with Acinetobacter strain culture was higher than in the treatment with *Enterobacter* strain culture (residual tea saponin contents, 3.813 ± 0.136 and 4.053 ± 0.023 mg/ml, respectively; *P* = 0.023) (Fig. 7D). There were no differences between the remaining cultures (*P* > 0.05) (Fig. 7D).

## DISCUSSION

The growth and performance of phytophagous insects feeding on different host plants are often mediated by plant secondary metabolites with antinutritive, deterrent, antimicrobial, and toxic effects (1, 5, 9). Feeding on suitable hosts facilitates phytophagous insects to successfully complete their life cycle; otherwise, they will encounter particularly strong pressure from defensive chemicals, especially for specialist species (1, 8, 25, 26). In nature, the level of plant-defensive chemicals differs with the species, depending on the plant genotype, growth conditions, and phenology (9). Insects feeding on low toxicity-level plants showed higher survival rates and body weights to some extent (24, 27). In this study, we monitored the larval performances in three different *Camellia* hosts. Examining the changes in larval weight according to age revealed that the larval development (weight and growth period) of CW was significantly affected by the host species. In agreement with previous reports, the larvae performed better in the seeds of *C. oleifera* with a low saponin content than in the seeds of the other two hosts, *C. reticulata* and C. sinensis, with higher saponin concentrations (Fig. 2 and 3). Therefore, the level of plant allelochemicals may restrict the range of host plants for phytophagous insects. Diverse and dense populations of microbes that can contribute to host phenotypes are crucial components of insects, as they mediate the insects’ ability to feed on a chemically defended plant (5, 28, 29). Microbes associated with the gut of phytophagous insects are subjected to a constant flow of plant toxins during digestion of plant material. In the present study, the variation in CW performance in seeds of different hosts, as indicated by differences in body weight (Fig. 2), might be caused by variation in the gut microbial community structure.

Plant allelochemicals exert a particularly strong selection pressure not only for herbivorous insects but also for their gut microbiota (28–32). Many species are colonized by microbes that have beneficial and fundamentally important impacts on host biology (33). The structure of microbial communities associated with the gut of insects is influenced by both host genotype and insect diet (7, 34, 35). For the first time, we have presented a complete gut bacterial characterization of a specialist insect, *Curculio chinensis,* feeding on the seeds of *Camellia* species (Fig. 4C and S6C). Each of the natural CW populations collected from the three tea trees, *C. oleifera*, C. sinensis, and *C. reticulata*, has considerably restricted gut bacterial microbiomes. The phyla *Proteobacteria*, *Firmicutes*, and *Bacteroidetes* comprised over 97% of the bacterial microbiome (Fig. 4C and S6C). All CW populations were dominated by *Proteobacteria* at the phylum level (Fig. 4C and S6C). Remarkably, analysis of OTU-level data showed that individual OTUs were not specific to a host population, but the gut microbiome of CW was influenced significantly by host diet (Fig. 4C and D and S6D and E), and the structure of the gut microbiome in CW feeding on the seeds of *C. reticulata* was significantly different from that of beetles feeding on the seeds of the other two host species (Fig. 3, 4C and D, and S6C and Table S1). The gut microbiome of CW larvae fed on *C. oleifera* and C. sinensis seeds showed obvious enrichment of *Proteobacteria* (>90%) (Fig. 1, 4C, and S6C). Nevertheless, the gut microbiome of weevils feeding on *C. reticulata* seeds was significantly enriched in *Firmicutes* and *Proteobacteria* (Fig. 1, 4C, and S6C). Although *Curculio chinensis* insects feeding on the three *Camellia* plants shared much of a common microbiome, they also harbored unique microbes (Fig. 4D). These results clearly illustrate the role of host diet in shaping bacterial microbiome composition in a seed predator and suggest that the feeding substrates of insects may harbor diet-specific bacterial communities to provide benefits to their hosts.

Recent studies revealed that some insect larvae (e.g., caterpillars, stinkbugs, and wood-feeding beetles) lack a resident gut microbiome and instead recruit beneficial bacteria from plant food or from the environment (33, 36–38). Dietary changes can rapidly alter gut microbial community structure (35, 39), and the induced changes in microbiome composition might confer an adaptive plasticity to insects that enhances their fitness in regard to the host plants presenting various levels of plant-defensive chemicals (5, 9). Therefore, the changes in the gut microbial community structure induced by plant toxins could be considered a first step resulting in insect specialization if suitable ecological conditions are satisfied. Gut microbes may be important in insect species-level diversification (5). Previous studies had already shown that CW exhibited significant phylogenetic variation related to *Camellia* host isolation (*C. oleifera*, C. sinensis, and *C. reticulata*) (18, 19). Our results show that the gut microbiomes of the camellia weevil varied significantly when the insects were fed on the seeds of the three *Camellia* species for a few months. However, the differences are smaller at shorter evolutionary time scales (35). Therefore, further experiments should take care to consider the bacterial community of the camellia weevil population interacting with each host at longer time scales in order to better understand microbiome-plant interactions.

Tea saponin has desirable characters such as strong foaming, emulsifying, dispersing, and wetting performance (40, 41), as well as anticancer (42), anti-inflammatory, antibacterial (42), and other biological activities (13), and it has been widely used as an insecticide in China (15). We herein demonstrated that there was a significant negative correlation between the body weight of CW larvae and tea saponin content in seeds during the same growth period (Fig. 2 and 3). Although high tea saponin content was detected in the seeds of both C. sinensis and *C. reticulata*, some larvae could still develop into adults, indicating that other factors may help *C. chinensis* resist tea saponins.

According to the analysis shown in the heat map and network diagram, Phenylobacterium, *Ochrobactrum*, *Erwinia*, Amycolatopsis, and Sediminibacterium spp. may play crucial roles in tea saponin metabolism (Fig. 5A and B). Some bacteria are known to detoxify saponin. For example, mixed cultures of Methanobrevibacter spp. and Methanosphaera stadtmanae in the crop of the avian foregut fermenter Opisthocomus hoazin were able to reduce the hemolytic activity of Quillaja saponins by 80% within a few hours (43). In the present study, 27 bacteria from the four genera *Enterobacter*, *Serratia*, Acinetobacter, and *Micrococcus* were isolated from the CW gut on medium containing tea saponin as a sole source of carbon and nitrogen (Fig. 7B). To evaluate whether the gut bacteria of CW can degrade tea saponin, we tested the rates of all cultured bacteria *in vivo*. Remarkably, our results illustrated the contribution of cultured bacteria toward tea saponin breakdown (Fig. 7D). Moreover, the degradation rate of a mixture of all cultured bacteria was higher than for any single isolate, and the Acinetobacter species showed strong degradation capacity (Fig. 7D). Finally, two bacteria, Acinetobacter calcoaceticus and Acinetobacter oleivorans, were identified to be involved in the degradation of tea saponin. A. calcoaceticus MTC 127 has been reported to have the ability to metabolize (+)-catechin (44). *A. oleivorans* DR1 has been demonstrated to modulate physiology and metabolism for efficient hexadecane utilization.

In conclusion, we presented a complete gut bacterial characterization of CW feeding on the seeds of *Camellia* species and demonstrated that the gut microbiome of CW was significantly influenced by the host diet. This framework may help us understand the ecology and functional impacts of microbe-host plant interactions. We also demonstrated that variation in CW performance in seeds of different hosts was affected by the saponin concentration in seeds. We showed that the microbiota was responsible for tea saponin degradation in the insect’s feeding. The gut bacteria of Acinetobacter facilitate CW in overcoming plant toxins. The results also provide novel avenues to develop environmentally friendly and sustainable strategies to control pest insects by regulating their gut microbial community structure.

## MATERIALS AND METHODS

### Insects for larval development monitoring.

More than 3,000 mature CW larvae were collected from organic *C. oleifera* orchards in Lishui, China (28°11′51.61ʺN, 120°23′15.25″E) in October 2017. All larvae were reared in sterile soil under controlled conditions (dark; soil temperature, 20°C; soil moisture, 15%) to obtain adults in the next year for the oviposition tests.

### Effect of fruit growth of different host plants on larval development.

To monitor the larval development in fruits of different *Camellia* hosts, 30 trees (at similar growth levels) were selected for each of three hosts, *C. oleifera*, C. sinensis, and *C. reticulata*, from insecticide-free orchards in Jinhua City (29°01′32.06″N, 119°37′28.45″E), China. To ensure that none of the fruits were naturally infested by camellia weevils, each tree was sealed with a transparent plastic mesh with one small zipper opening during April 2018 (before CW adults emerged out of the soil), thus preventing access to wild females. In late May, 10 1-day-old CW adults (female-to-male ratio, 1:1) were randomly selected from several hundred mass-reared adults, as described above, and freely reared in each mesh for mating and oviposition. From the end of July to the middle of September, maturing fruits (*n* = 100) were picked randomly from the 30 mesh-sealed trees for each host plant every 10 days, and developing larvae were extracted from the fruits and weighed synchronously. After the larvae were removed from the fruits, undamaged seeds were collected for chemical analysis.

### Tea saponin content analysis.

Using a study by Zhang et al. (45) as a reference, the chromatographic conditions involved the Eclipse XDB-C_18_ (4.6 mm by 250 mm, 5 μm; Agilent Technologies, Inc.) chromatographic column and a mobile phase of methanol-water (V_methanol_:V_water_ = 9:1). The detection wavelength was 210 nm. The column temperature was 25°C. For the standard treatment, the tea saponin standard was weighed at 0.5 g (accurate to 0.0001 g), diluted with methanol using ultrasound, and adjusted to a volume of 100 ml in a volumetric flask. Then, 1.00, 3.00, 5.00, 7.00, and 9.00 ml were removed and dissolved in 5 (50-ml) volumetric flasks in a constant volume of methanol. After filtration through a 0.45-μm microporous membrane, the regression equation was obtained by using the mass concentration as the *x* coordinate and the corresponding peak area as the *y* coordinate. For determination of the content of tea saponin in samples, 0.058 g (accurate to 0.0001 g) of tea saponin was sampled using an analytical balance and then dissolved in methanol via ultrasound, and a fixed volume was placed in a 50-ml volumetric flask. Filtration through a 0.45-μm microporous membrane was carried out by high-performance liquid chromatography (HPLC). The formula for calculating the content of tea saponin is shown in the following equation: tea saponin content (%) = X × V/m × 100, where X is the concentration of the sample solution (g/ml), V is the volume of the sample at constant volume (ml), and m is the mass of the sample (g).

### Effects of resistant active substances on the diversity of microbiota.

One thousand mature CW larvae were collected from *C. oleifera* trees in the autumn of 2018. In the laboratory, all of the specimens were soaked in alcohol for 1 min; surface debris was cleaned with an ultrasonic wave, and then the intestines were dissected with phosphate buffer. The collected intestines were placed in a homogenizer, and the ratio of the intestines to the buffer of phosphoric acid was 1:4 (Fig. 1, step 1). After the homogenizing process, the suspension was stored at 4°C for 1 day. Before the homogenizing process, the target-resistant active substances (tea saponin, EGCG, caffeine, tannic acid, and theanine) were added to the nutrient agar (NA) medium, and the concentration of each substance was set according to the previous determination in the plant from the highest concentration to 0. Tea saponin and tannic acid concentrations ranged from 5 mg/g to 100 mg/g, containing 20 equidifferent concentration gradients; EGCG, caffeine, and theanine concentrations ranged from 0.3 mg/g to 6 mg/g, with 20 equidifferent concentration gradients. Each chemical treatment was repeated five times. The NA medium was set as a control (CK) with five replicates (Table S3). Four milliliters of prepared liquid medium was taken, and 40 μl of intestinal suspension was added to the medium (Fig. 1, step 2). All samples and CK (NA medium without any target-resistant active substance) were cultured under 37°C at 200 rpm for 24 h (Fig. 1, step 3), and then all the bacterial solution samples were stored at −80°C for testing. Each sample had five replicates.

### DNA extraction, PCR amplification, and high-throughput sequencing.

DNA extraction was carried out using a modified cetyltrimethylammonium bromide (CTAB) protocol (46), with a minor alteration in incubation time (to 12 h). Negative controls (extraction without feces) were included to monitor possible contamination for each batch of DNA extraction. The extracted DNA was used as the template for amplification of the V3-V4 variable region of the bacterial 16S rRNA gene, which has high sequence coverage for prokaryotes and produces an appropriately sized amplicon, as a barcode primer for Illumina sequencing (47). Both primers contained Illumina adapters, and the reverse primer contained a 12-bp barcode sequence unique to each sample. The PCR amplification was carried out in a total reaction volume of 25 μl with three replicates for each sample. PCR amplification was performed under the following conditions: initial denaturation at 94°C for 1 min, followed by 30 cycles of 94°C for 20 s, 57°C for 25 s, and 68°C for 45 s, ending at 68°C, with a final extension step of 10 min. All PCR amplifications were performed in triplicate and then combined. PCR amplicons were then pooled in equimolar concentrations on a 1% agarose gel, and purified PCR products were recovered using a GeneJET gel extraction kit. High-throughput sequencing of the PCR products was performed on an Illumina MiSeq platform (MiSeq PE250) at Shanghai Personal Biotechnology Co., Ltd. (Shanghai, China).

### 16S rRNA sequence data processing and analysis.

The Quantitative Insights into Microbial Ecology (QIIME, v1.8.0) pipeline was employed to process the sequencing data, as previously described (48). Briefly, raw sequencing reads with exact matches to the barcodes were assigned to respective samples and identified as valid sequences. All raw 16S sequences were quality trimmed using Cutadapt (v1.9.1; https://cutadapt.readthedocs.io/en/stable/) (49) and assigned to their respective samples according to the unique nucleotide barcodes (http://www.drive5.com/usearch/manual/chimeras.html). After the removal of barcodes and primers, paired-end sequences were merged and quality filtered using the UCHIME algorithm (http://www.drive5.com/usearch/manual/uchime_algo.html) (50). We generated a total of 42,871,626 high-quality sequences from 18 samples (averaging 69,807 and ranging from 40,742 to 1,256,539 reads per sample). We analyzed high-quality reads with UNOISE, discarded low-abundance OTUs (<8 total counts), and obtained 1,089 OTUs. Rarefaction curves of all 18 samples for 16S rRNA gene sequencing tended to be saturated (Fig. S4), indicating that the sequencing depths for these specimens were appropriate.

10.1128/mSystems.00692-19.3 FIG S3 Heat map shows the Pearson correlation coefficient of the relative abundances of all samples. Download FIG S3, PDF file, 1.4 MB. Copyright © 2020 Zhang et al. 2020 Zhang et al. This content is distributed under the terms of the Creative Commons Attribution 4.0 International license.

10.1128/mSystems.00692-19.4 FIG S4 Similar trends reflected by pairwise correlations between treatment concentrations in the samples receiving one of the five treatments. Download FIG S4, PDF file, 0.1 MB. Copyright © 2020 Zhang et al. 2020 Zhang et al. This content is distributed under the terms of the Creative Commons Attribution 4.0 International license.

10.1128/mSystems.00692-19.5 FIG S5 Heat maps showing the relative abundances of some selected species across the samples receiving one of the five treatments. Download FIG S5, PDF file, 0.1 MB. Copyright © 2020 Zhang et al. 2020 Zhang et al. This content is distributed under the terms of the Creative Commons Attribution 4.0 International license.

10.1128/mSystems.00692-19.6 FIG S6 The gut microbial community analysis of the CW larvae feeding on the three host plants. (A and B) Rarefaction curve of 16S rRNA in the gut microbiome of *Curculio chinensis* Chevrolat on the three host plants. (C) Heat map showing the relative abundances of some selected species across *Curculio chinensis* Chevrolat samples. LEfSe showing comparison of *Curculio chinensis* Chevrolat samples between three host plants at all levels. (D) The module plots the biomarkers found by LEfSe, ranking them according to their effect size and associating them with the class with the highest median. (E) This module produces cladograms representing the LEfSe results on the hierarchy induced by the label names. Red blocks represent significant difference at the genus level. Blue blocks represent significant differences in *Proteobacteria*. Download FIG S6, PDF file, 1.3 MB. Copyright © 2020 Zhang et al. 2020 Zhang et al. This content is distributed under the terms of the Creative Commons Attribution 4.0 International license.

Sequence data analyses were mainly performed using QIIME (48) and the R packages (v3.5.2) (51). These sequences were clustered into OTUs with a sequence threshold of 97% similarity by UPARSE (52), and representative sequences of OTUs were picked up simultaneously. The singletons and chimeras were removed during the UPARSE procedure. Taxonomic assignment of 16S rRNA representative sequences was carried out with mothur and the SILVA classifier (https://www.arb-silva.de/) (53) based on the SSUrRNA database, and sequences (OTUs) assigned to MUSCLE (version 3.8.31; http://www.drive5.com/muscle/) were aligned for subsequent analysis (54). Resampled 16S rRNA OTU subsets (15,000 sequences per sample) were used to calculate alpha diversity and beta diversity. In this research, we calculated four kinds of alpha diversity to measure the biodiversity of the community in the gut of beetles and in bacteria cultured *in vitro*. Richness was obtained by counting the observed species numbers associated with rarefaction curves. Alpha-diversity indices (ACE, Chao1, Shannon, and inverse Simpson) were calculated according to species abundance using the vegan package in R (v.3.2.5) (51). Statistical analyses of differentially abundant OTUs were performed using the edgeR library by fitting a negative binomial generalized linear model to the OTUs (55). A phylogenetic tree was constructed with QIIME. UniFrac was carried out with the phylogenetic tree to perform phylogenetic beta-diversity analysis (56). The differences in the beta diversity indices of bacterial communities were determined by PCoA. Student's *t* test was employed to determine whether the distances between community compositions and distributions of five treatments were significantly different, using SPSS. Correlations between community composition and distributions of tolerance with the five treatments were tested using Mantel tests in R (v. 3.5.1) between Bray-Curtis distance matrices of community composition and Euclidean distance matrices of trait distributions (12, 51). The Pearson correlation coefficient was calculated using the mean of all microbiota replicates from each condition at each point and was visualized by using the ggcorrplot package (57). A comparison of the microbiota was performed by an Adonis function in the VEGAN package. Permutational multivariate analysis of variance (PERMANOVA) and analysis of similarity (ANOSIM) were carried out to test whether the gut microbiome structure was significantly different between two sites using a method implemented in the R package VEGAN (58). Linear discriminant analysis (LDA) effect size (LEfSe) analysis was performed using the LEfSe tool to identify specialized bacterial groups within each type of sample (59). The Kruskal-Wallis rank sum test was used to detect significantly different abundances and generate LDA scores to estimate the effect size (threshold, ≥4.0) in the LEfSe analysis.

We employed the following method to predict the molecular functions of each sample based on 16S rRNA sequencing data. We used the KEGG database and performed closed-reference OTU picking using the sampled reads against a Greengenes reference taxonomy (v.13.5). The 16S rRNA copy number was then normalized. After that, molecular functions were predicted, and the final data were summarized into KEGG pathways. The mothur software was used to calculate all possible pairwise Spearman rank correlations of the abundance in the top 50 genera. A correlation was considered to be valid if the absolute value of the Spearman rank correlation coefficient was both ≥0.6 and statistically significant (*P* < 0.01), and all the values were imported into the Cytoscape (https://cytoscape.org/) software for analysis (60). The effects of five treatments with active substances on the microbial diversity of CW gut microbiome data were visualized using the Circos software (http://circos.ca/) (61).

### Determination of tea saponin content and isolation of tea-saponin-degrading bacteria and their identification.

Three hundred fresh fruits were collected from May to September, transported to the laboratory, and rapidly split, and the kernels were extracted to determine the content of tea saponins (45). The program PICRUSt (http://huttenhower.sph.harvard.edu/galaxy/root?tool_id=PICRUSt_normalize) was used for predicting microbial metabolism (62). Using the R software, cluster analysis was performed, and a heat map of the top 50 functional groups was constructed. In order to better identify the key OTUs, we used a Manhattan diagram to display the differences between OTU and taxonomy. The R scripts required for the computational analyses performed in this research were altered based on https://github.com/microbiota/Zhang2019NBT (48). According to the experimental results of the effects of tea saponin treatment on the diversity of bacterial flora, treatment with 1 to 40 mg/g tea saponin could be selected as the initial concentration for verifying the tea saponin metabolism ability of microbiota. Five grams of tea saponin (99% purity), 5 g (NH4)_2_SO_4_, 2.5 g Na_2_CO_3_, 0.3 g KH_2_PO_4_, 0.05 g FeSO_4_·7H_2_O, and 0.5 g MgSO_4_ were added into 1,000 ml pure water to prepare the medium for tea saponin as a single carbon source. All multistrain and single-strain cultures were kept at 37°C and 200 rpm. The absorbance at 620 nm of all culture media was adjusted to 1.59 ± 3 (after dilution by 10^−11^ fold; 30 ± 5 strains were grown on solid NA medium). At the same time, solid medium containing different concentrations of tea saponin (5 mg/ml) was also used to verify the bacteriostatic test of tea saponin under 37°C for 24 h to isolate single strains with a given metabolic function. Then, single strains were selected and cultured in liquid NA medium under aseptic conditions. One milliliter of multistrain and single-strain cultures was absorbed into the 1,000-ml medium with tea saponin as the sole carbon source. Samples were taken every 12 h and repeated three times, for measuring the residual concentration of tea saponin by liquid chromatography (45).

### Data availability.

The raw sequence data of the CW gut microbiome samples from each species of *Camellia* (*C. oleifera*, C. sinensis, and *C. reticulata*) and five strains of bacteria cultured *in vitro* were uploaded to GenBank SRA with the accession numbers SAMN11982877 to SAMN11982894 (larvae) and SAMN11998270 to SAMN11998472 (*in vitro* bacteria).
