# Supplementary figures and images for: The Gut Microbiota in Camellia Weevils Are Influenced by Plant Secondary Metabolites and Contribute to Saponin Degradation
Source: mSystems. 2020 Mar 17;5(2):e00692-19. doi: 10.1128/mSystems.00692-19 (PMC7380582; doi:10.1128/mSystems.00692-19)

# Tea saponin

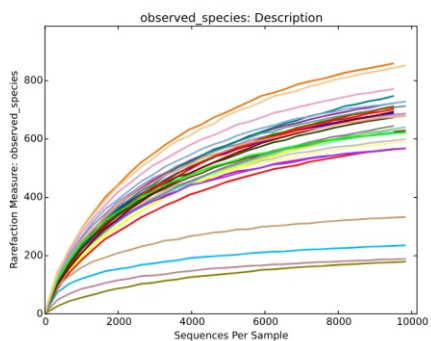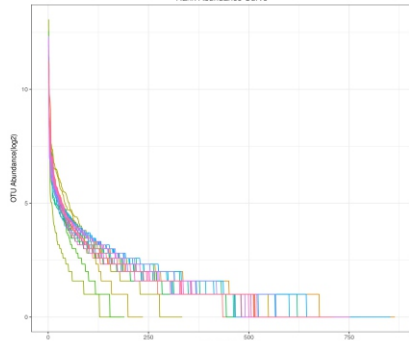

# Tannin acid

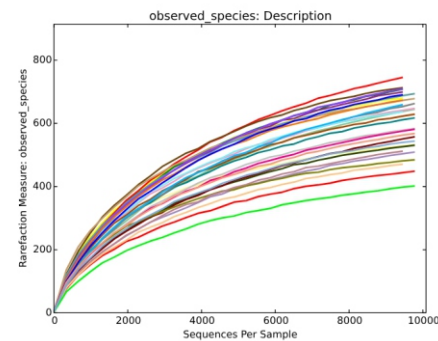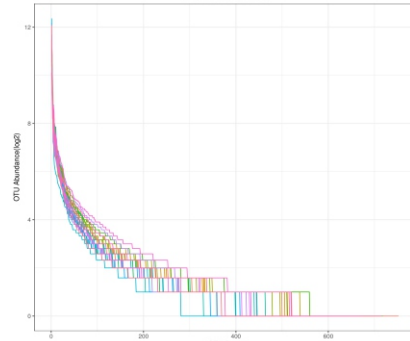

# EGCG

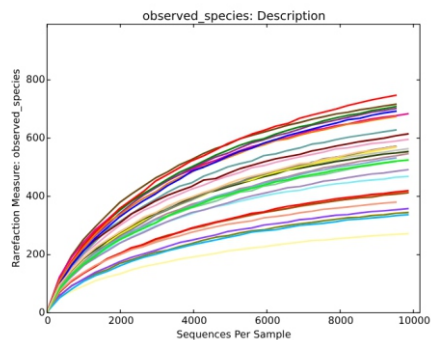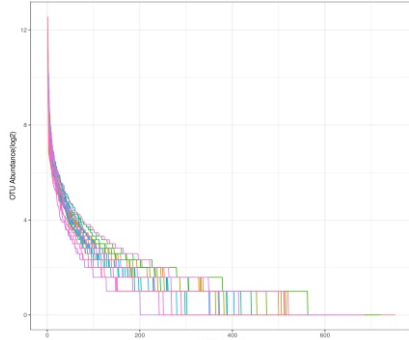

# Caffeine

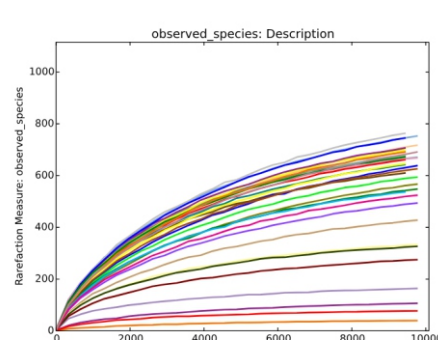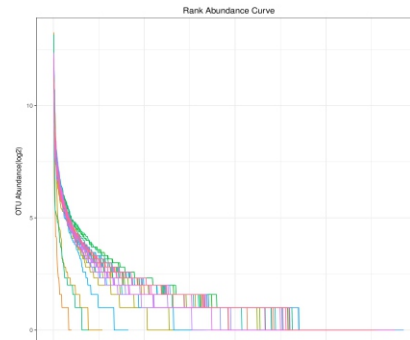

# Theanine

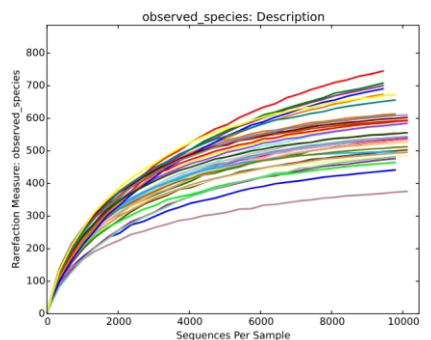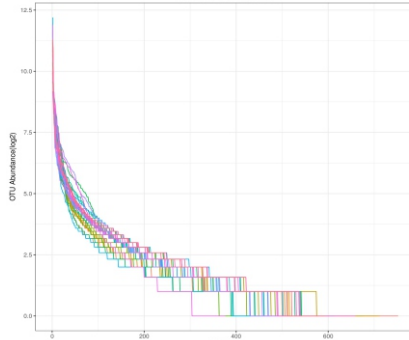

Supplement: FIG S1 [file mSystems.00692-19-sf001.pdf]

class

Tea saponin

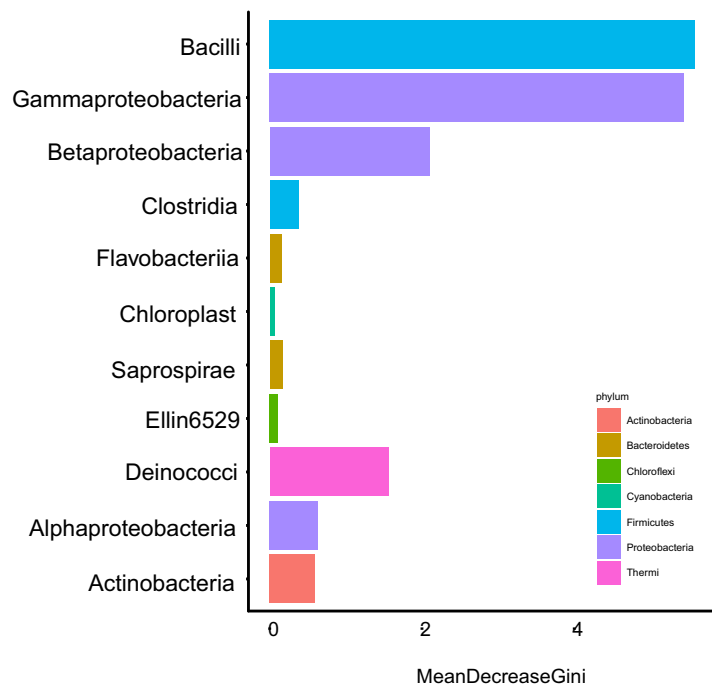

EGCG

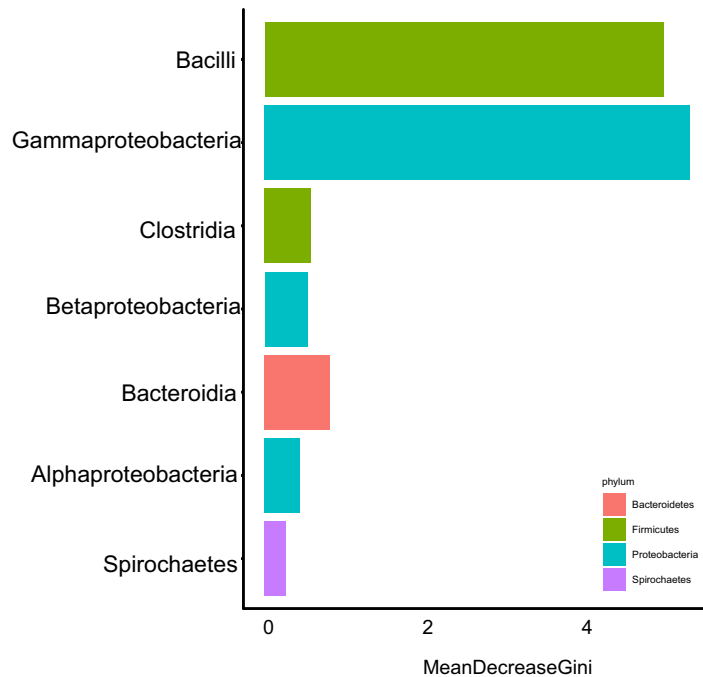

Tannin acid

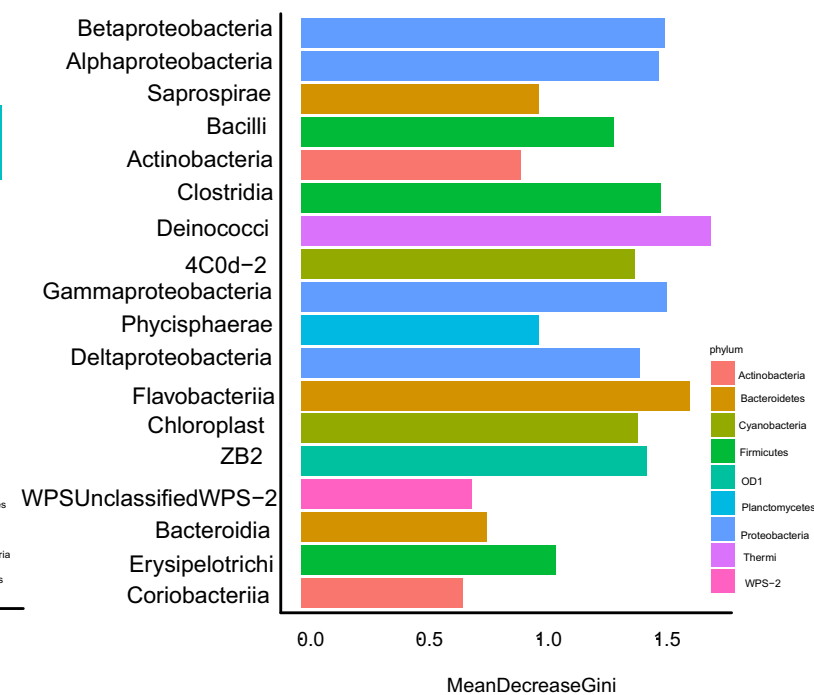

Caffeine

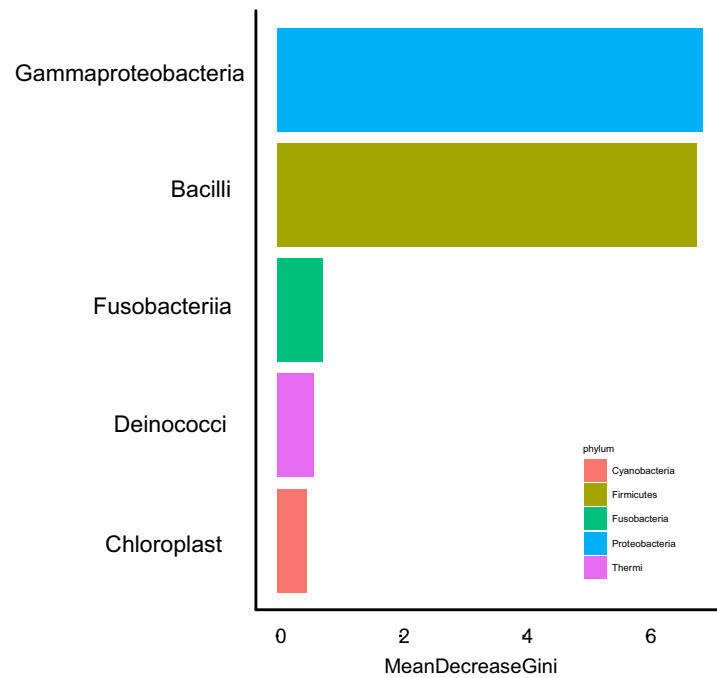

Theanine

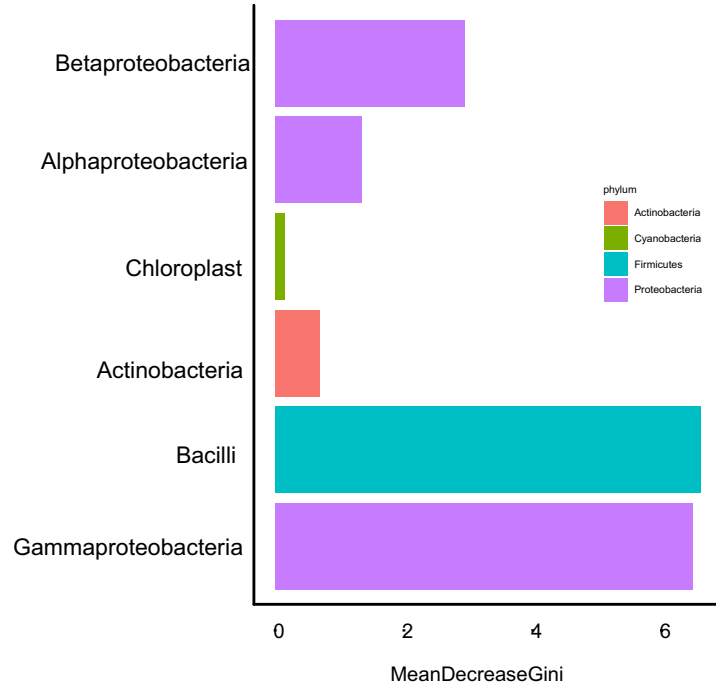

Supplement: FIG S2 [file mSystems.00692-19-sf002.pdf]

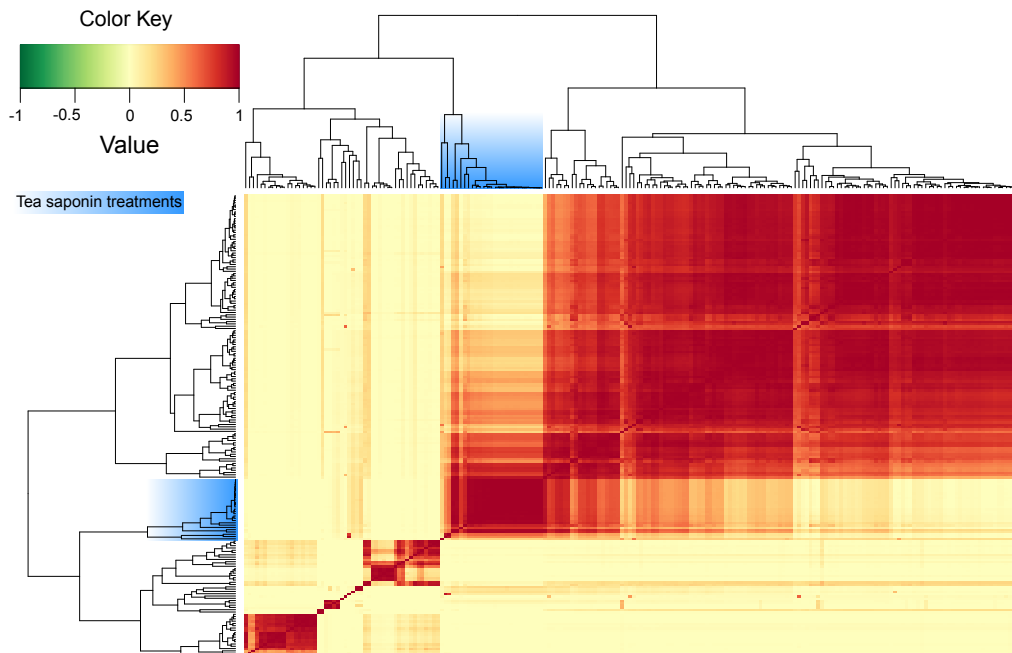

Supplement: FIG S3 [file mSystems.00692-19-sf003.pdf]

Tea saponin

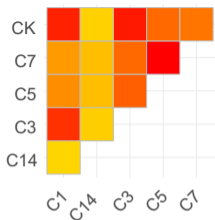

EGCG

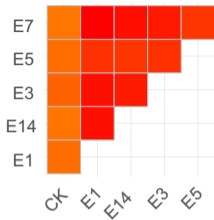

Tannin acid

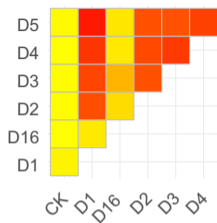

Caffeine

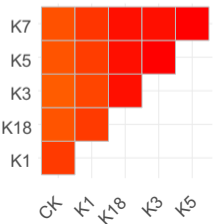

Theanine

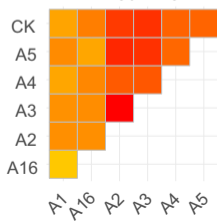

Corr

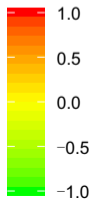

Supplement: FIG S4 [file mSystems.00692-19-sf004.pdf]

Tea saponin

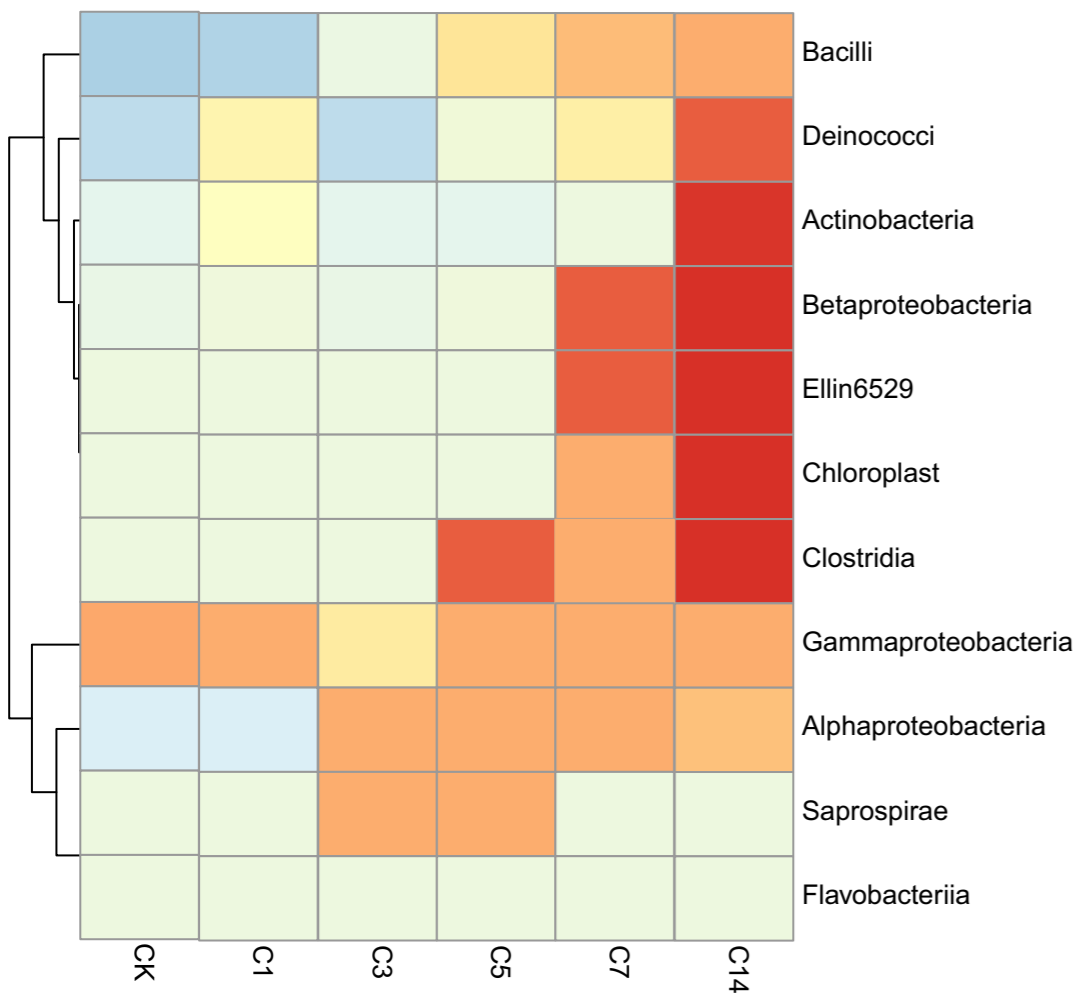

EGCG

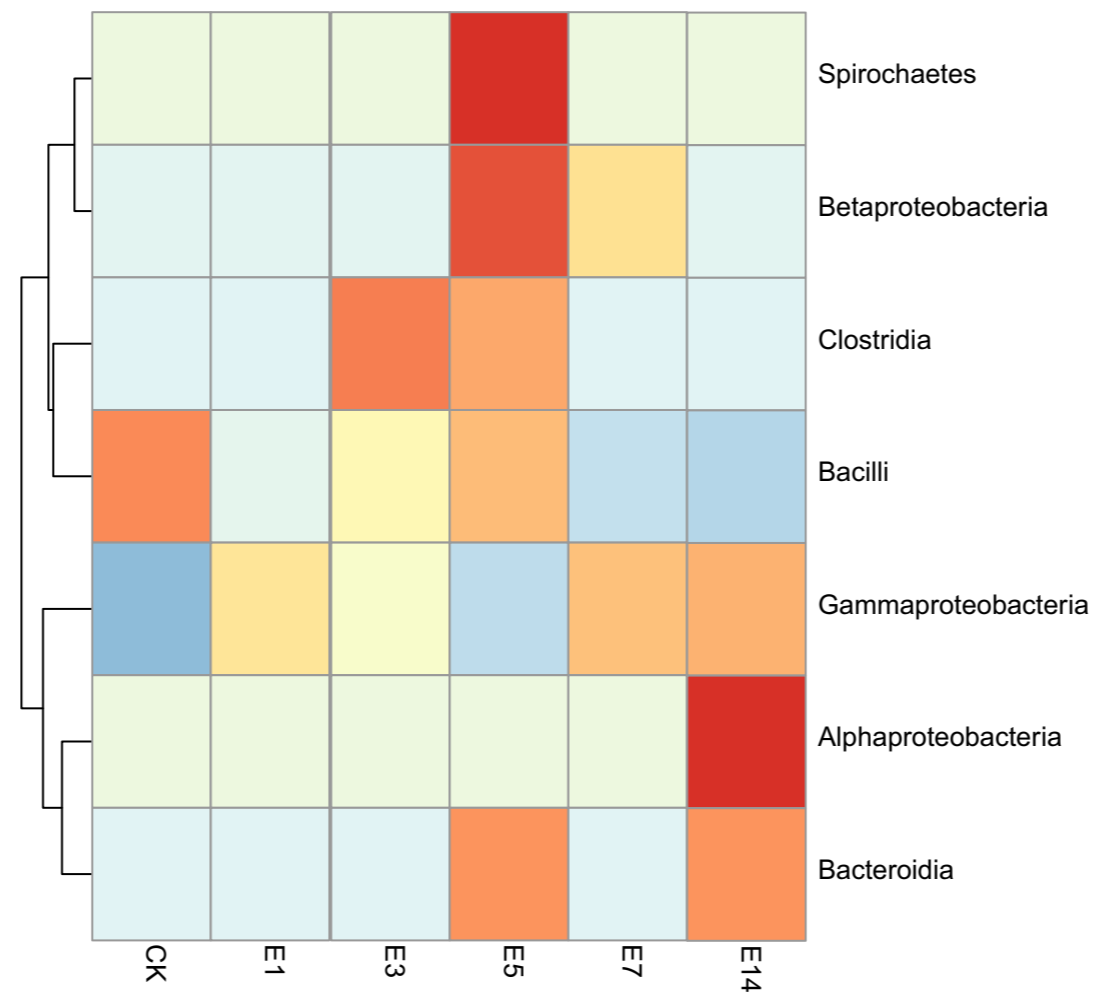

Tannin acid

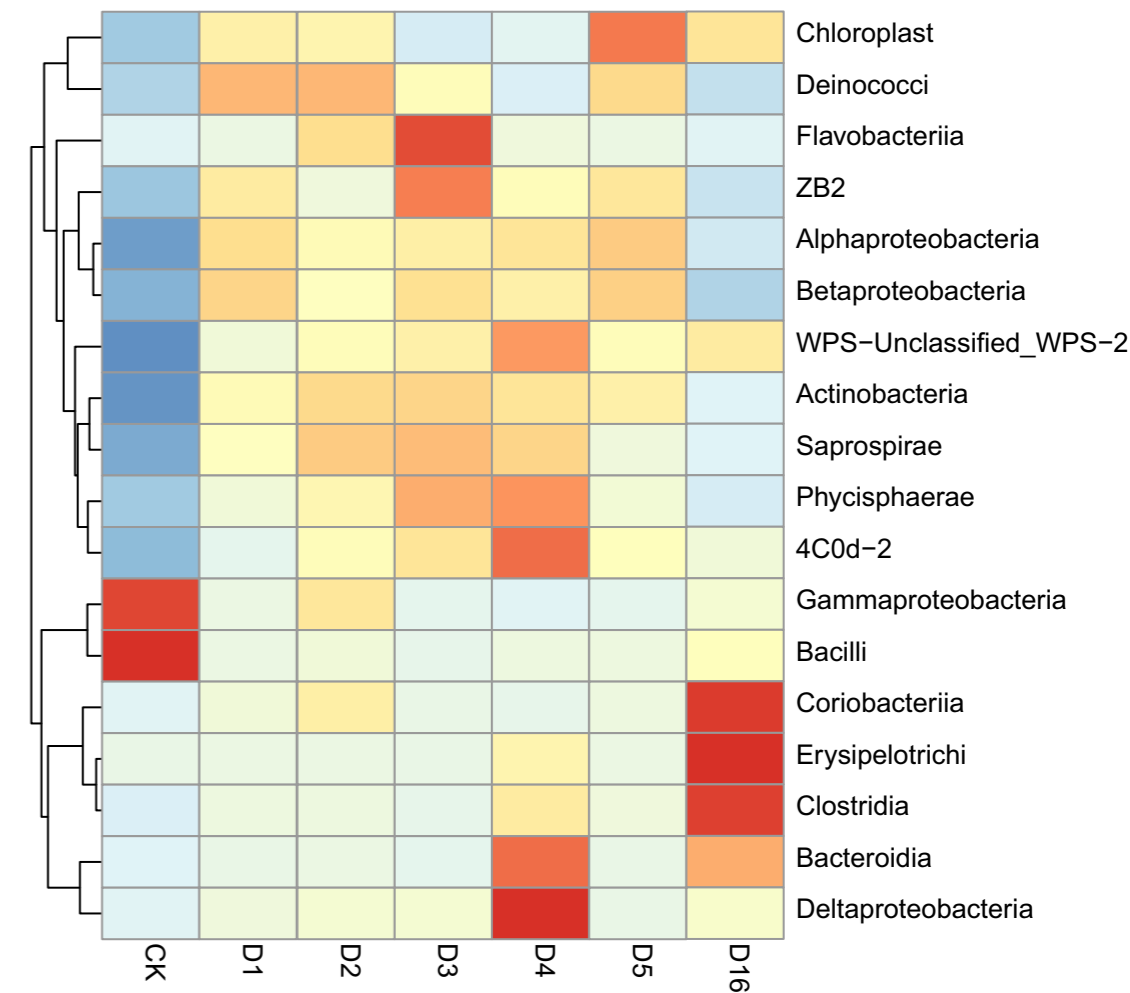

Caffeine

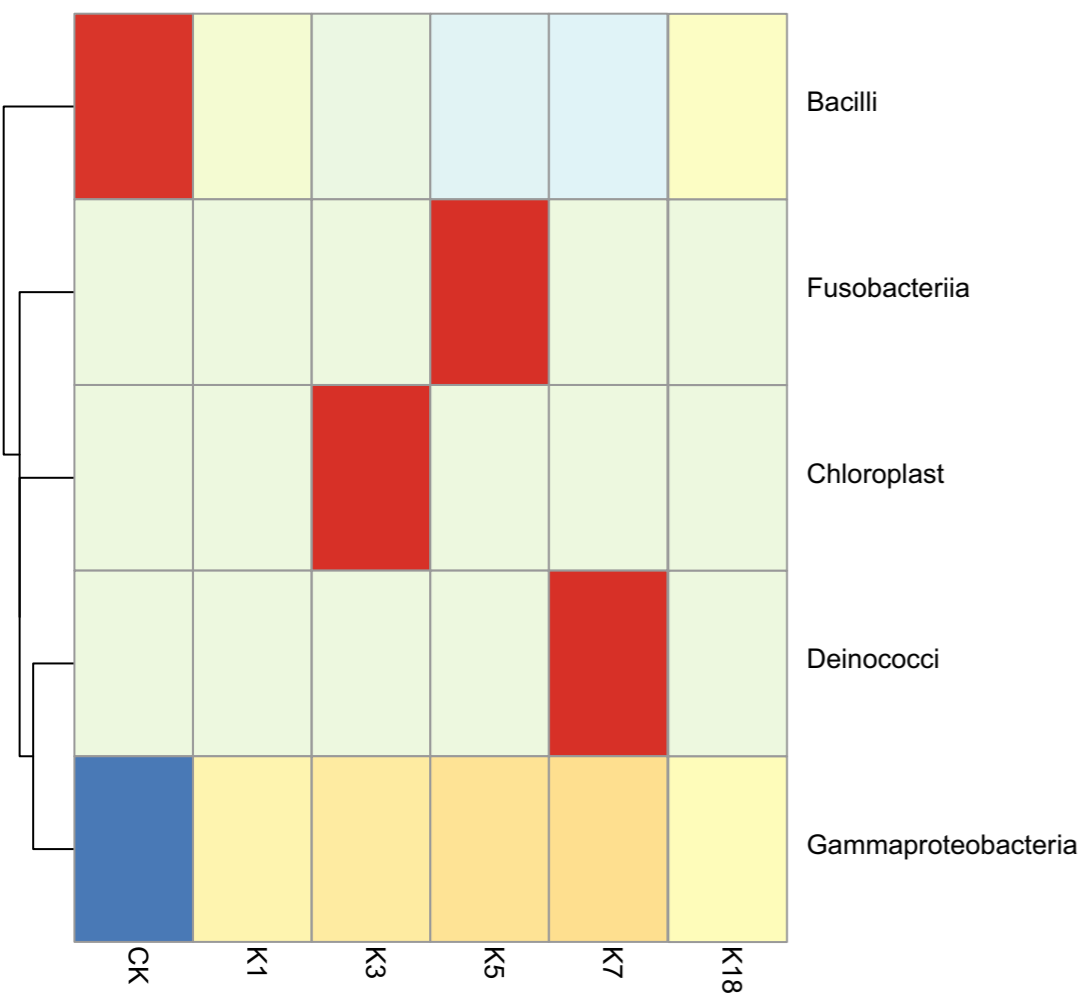

Theanine

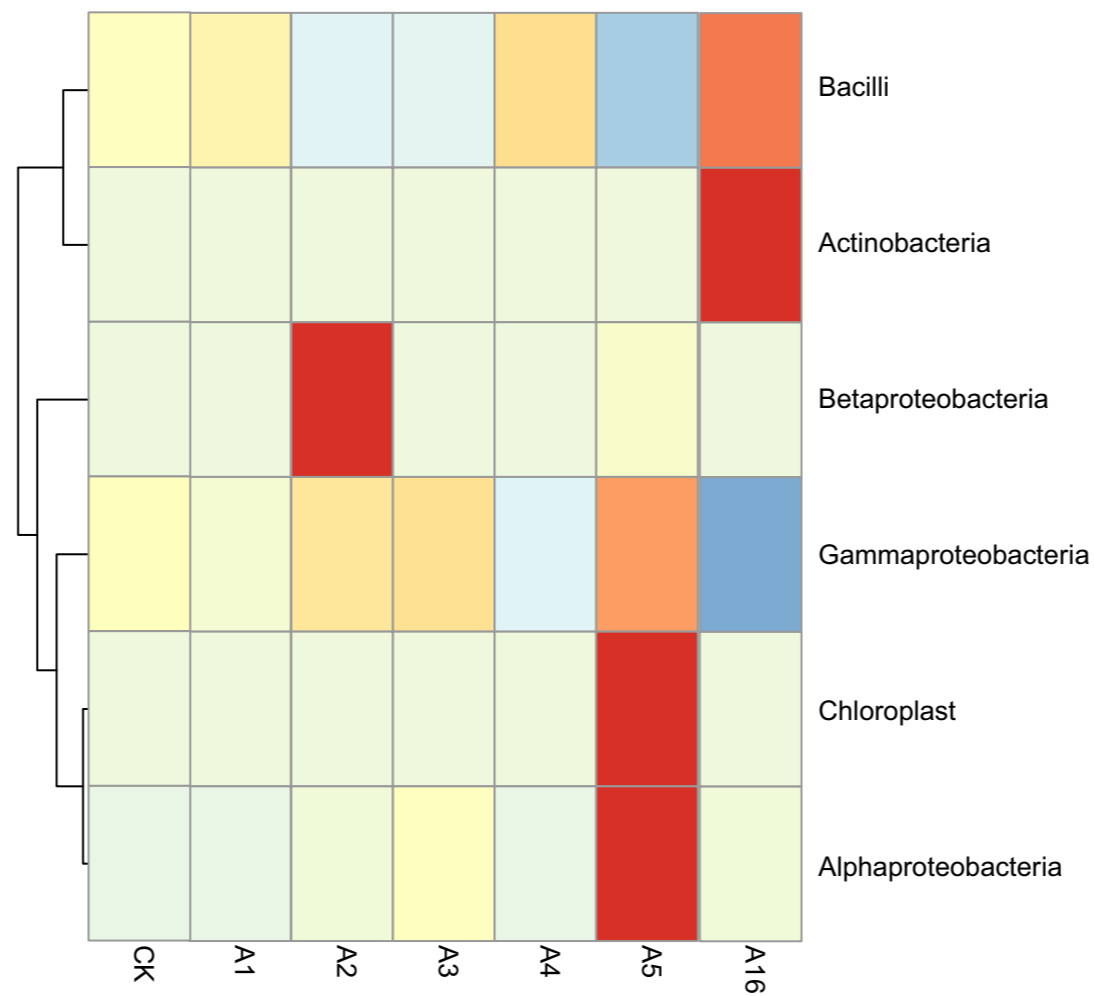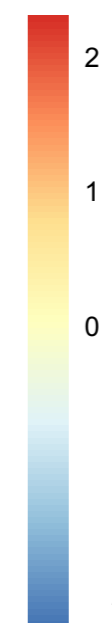

Supplement: FIG S5 [file mSystems.00692-19-sf005.pdf]

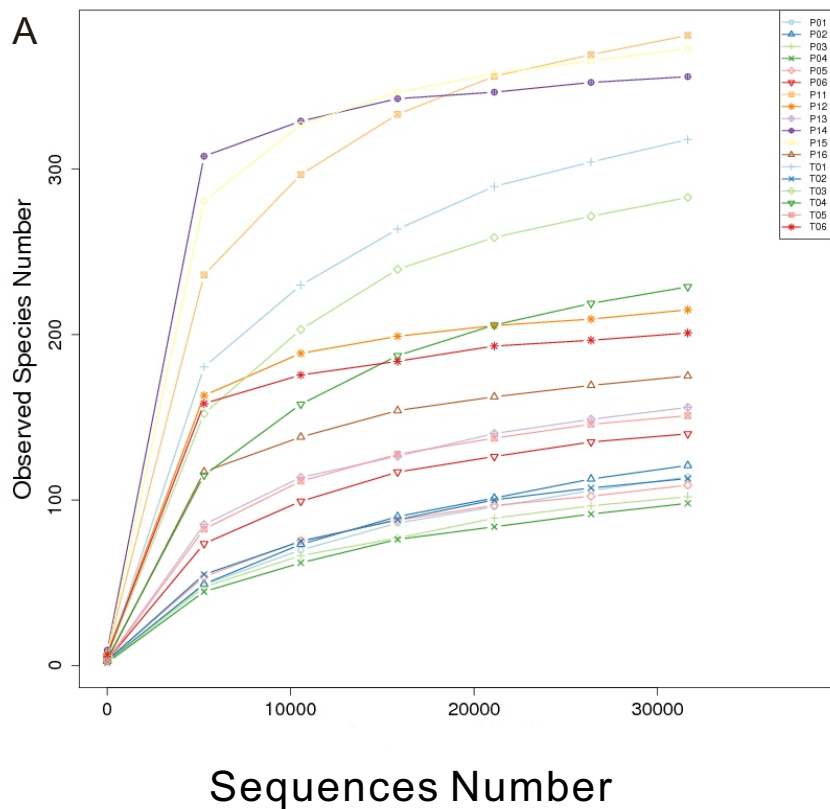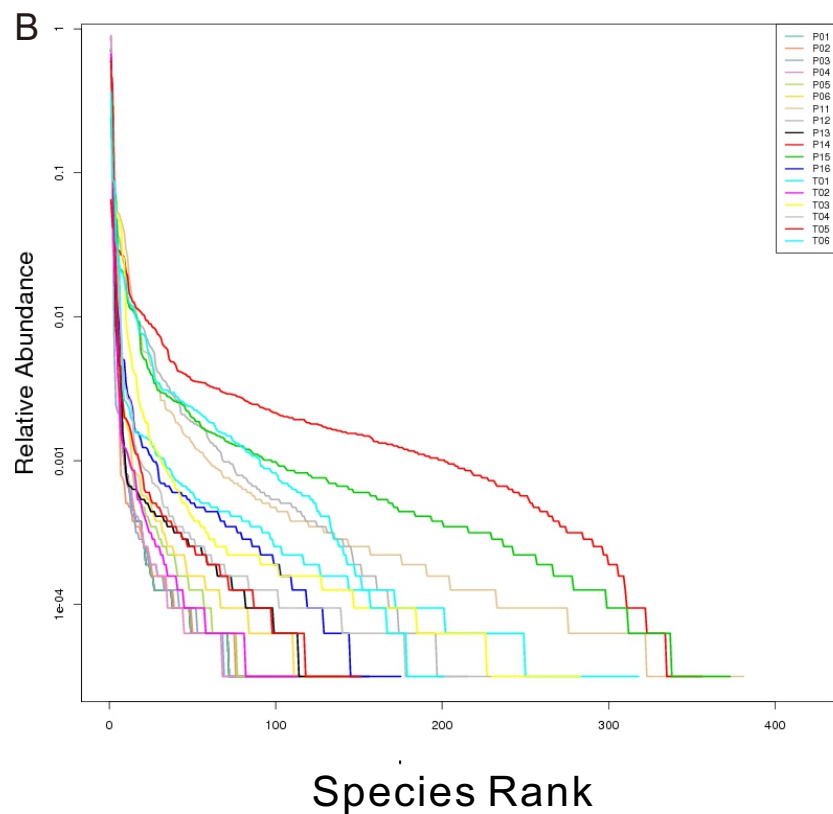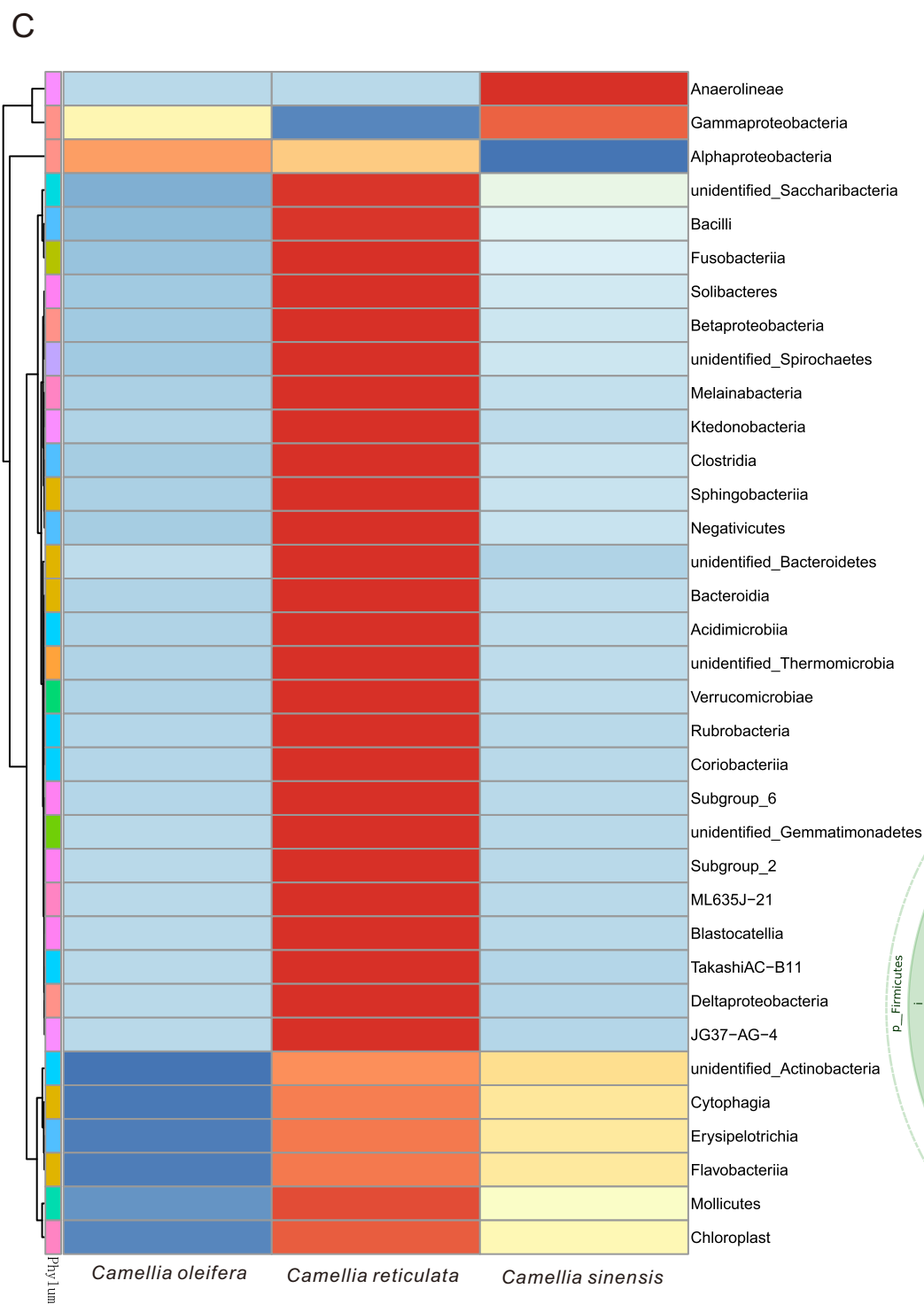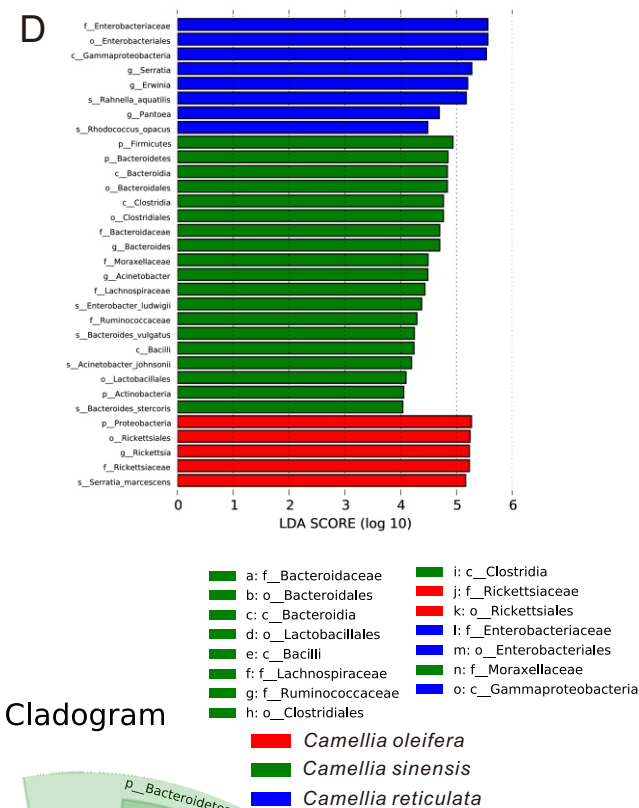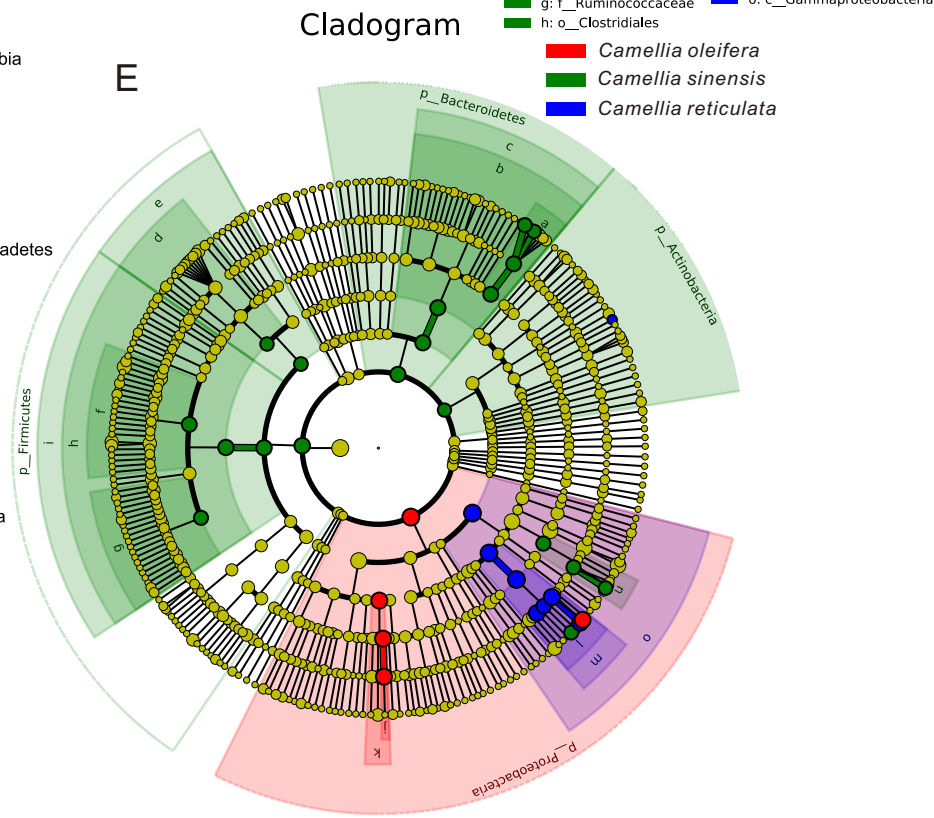

Supplement: FIG S6 [file mSystems.00692-19-sf006.pdf]
