# Supplementary material for: The Gut Microbiota in Camellia Weevils Are Influenced by Plant Secondary Metabolites and Contribute to Saponin Degradation
Source: mSystems. 2020 Mar 17;5(2):e00692-19. doi: 10.1128/mSystems.00692-19 (PMC7380582; doi:10.1128/mSystems.00692-19)
Supplement: TABLE S1 [file mSystems.00692-19-st001.docx]

| Dissimilarity tests | ANOSIM | | PERMANOVA | |
| --- | --- | --- | --- | --- |
|  | R | Significance | R^2^ | Significance |
| *C. oleifera* - *C. sinensis* | 0.5907 | 0.001** | 0.23539 | 0.001** |
| *C. reticulata* - *C. oleifera* | 0.9 | 0.003** | 0.33751 | 0.003** |
| *C. reticulata* - *C. sinensis* | 0.3963 | 0.017* | 0.16544 | 0.077 |
