# Supplementary material for: The Gut Microbiota in Camellia Weevils Are Influenced by Plant Secondary Metabolites and Contribute to Saponin Degradation
Source: mSystems. 2020 Mar 17;5(2):e00692-19. doi: 10.1128/mSystems.00692-19 (PMC7380582; doi:10.1128/mSystems.00692-19)
Supplement: TABLE S2 [file mSystems.00692-19-st002.docx]

|  | Modularity | Clustering coefficient | Graph density | Average degree | Average path length |
| --- | --- | --- | --- | --- | --- |
| *C. oleifera* | 0.397429 | 0.569646 | 0.231963 | 64.94964 | 1.823585 |
| *C. sinensis* | 0.434348 | 0.644556 | 0.244801 | 144.922 | 1.801522 |
| *C. reticulata* | 0.460424 | 0.670918 | 0.254562 | 129.8268 | 1.804799 |
