# Supplementary material for: The Gut Microbiota in Camellia Weevils Are Influenced by Plant Secondary Metabolites and Contribute to Saponin Degradation
Source: mSystems. 2020 Mar 17;5(2):e00692-19. doi: 10.1128/mSystems.00692-19 (PMC7380582; doi:10.1128/mSystems.00692-19)
Supplement: TABLE S3 [file mSystems.00692-19-st003.docx]

| Model | Spearman | | Kendall | | Pearson | |
| --- | --- | --- | --- | --- | --- | --- |
|  | r | *P* | r | *P* | r | *P* |
| Tea saponin | 0.3749 | 0.001*** | 0.2785 | 0.001*** | 0.2747 | 0.002*** |
| EGCG | −0.1504 | 0.932 | −1.04 | 0.938 | −0.1185 | 0.865 |
| Caffeine | −0.1038 | 0.889 | −0.0708 | 0.892 | −0.1017 | 0.892 |
| Tannic acid | 0.2163 | 0.04* | 0.1283 | 0.09 | 0.3314 | 0.01* |
| Theanine | 0.275 | 0.017** | 0.1923 | 0.017** | 0.2857 | 0.027** |
