# Supplementary material for: The Gut Microbiota in Camellia Weevils Are Influenced by Plant Secondary Metabolites and Contribute to Saponin Degradation
Source: mSystems. 2020 Mar 17;5(2):e00692-19. doi: 10.1128/mSystems.00692-19 (PMC7380582; doi:10.1128/mSystems.00692-19)
Supplement: TABLE S4 [file mSystems.00692-19-st004.docx]

| Wilcox tests | ACE | | chao1 | | Shannon | | Simpson | |
| --- | --- | --- | --- | --- | --- | --- | --- | --- |
|  | Dif. | Signifcance | Dif. | Signifcance | Dif. | Signifcance | Dif. | Signifcance |
| *C.oleifera - C. reticulata* | -9.666667 | 0.0003 *** | -9.333333 | 0.0007*** | -9.166667 | 0.001*** | -7.333333 | 0.0148* |
| *C. reticulata - C.sinensis* | -6.833333 | 0.0045** | -6.166667 | 0.0128* | -5.833333 | 0.020* | -4.666667 | 0.1 |
| *C. oleifera - C. sinensis* | 2.8333 | 0.1761* | 3.166667 | 0.1672 | 3.333333 | 0.158 | 2.666667 | 0.3326 |
